# Cyclometalated iridium–BODIPY ratiometric O_2_ sensors†
†Electronic supplementary information (ESI) available: Crystallographic summary tables, NMR spectra for all new compounds, additional UV-vis absorption data, high-resolution mass spec data, and additional photoluminescence spectra. CCDC 1883526–1883528. For ESI and crystallographic data in CIF or other electronic format see DOI: 10.1039/c9sc00696f


**DOI:** 10.1039/c9sc00696f

**Published:** 2019-04-15

**Authors:** Ku Sun Choung, Karen Marroquin, Thomas S. Teets

**Affiliations:** a University of Houston , Department of Chemistry , 3585 Cullen Blvd., Room 112 , Houston , TX 77204-5003 , USA . Email: tteets@uh.edu

## Abstract

Here we introduce a new class of ratiometric O_2_ sensors for hypoxic environments. Two-component structures composed of phosphorescent cyclometalated Ir(iii) complexes and the well-known organic fluorophore BODIPY have been prepared by the 1 : 1 reaction of bis-cyclometalated iridium synthons with pyridyl-substituted BODIPY compounds. Two different cyclometalating ligands are used, which determine the relative energies of the iridium-centered and BODIPY-centered excited states, and the nature of the linker between iridium and BODIPY also has a small influence on the photoluminescence. Some of the conjugates exhibit dual emission, with significant phosphorescence from the iridium site and fluorescence from the BODIPY, and thus function as ratiometric oxygen sensors. Oxygen quenching experiments demonstrate that as O_2_ is added the phosphorescence is quenched while the fluorescence is unaffected, with dynamic ranges that are well suited for hypoxic sensing (*p*O_2_ < 160 mmHg).

## Abstract 2

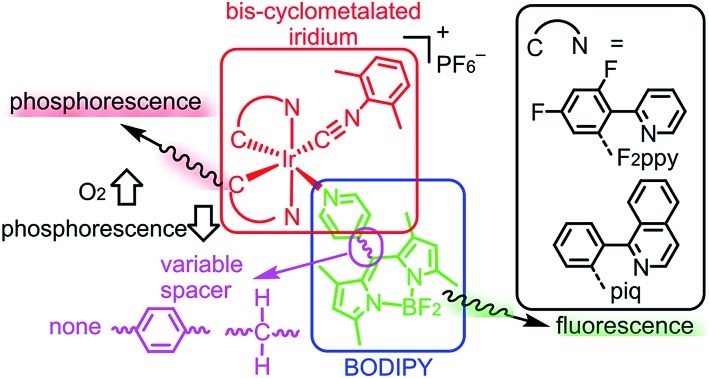
Cyclometalated iridium–BODIPY conjugates are prepared by a simple strategy and are effective ratiometric sensors for molecular oxygen.

## Introduction

Molecular oxygen plays a major role in a large range of chemical and biochemical reactions essential to aerobic metabolism.^1^–^3^ As hypoxia is associated with a variety of diseases in our daily lives and also found in tumor cells,^4^ many researchers have worked to develop reliable methods for sensing triplet oxygen in the past several years.^5^–^10^ Accurate O_2_ sensors are especially important in cancer biology, where the O_2_ level in tumor cells can be used to determine the tumor's metabolic state and guide therapeutic protocols. Ratiometric luminescent oxygen sensors, where the readout is a ratio of two emission signals, provide an advantage over conventional sensors in that they circumvent the need to measure absolute emission intensities as the primary readout. This ratiometric response minimizes detection errors resulting from heterogeneous cellular environments, differences in excitation power, or variations in optical path length, and as such is a considerably more reproducible method for measuring cellular O_2_.^11^

**Fig. 1:**
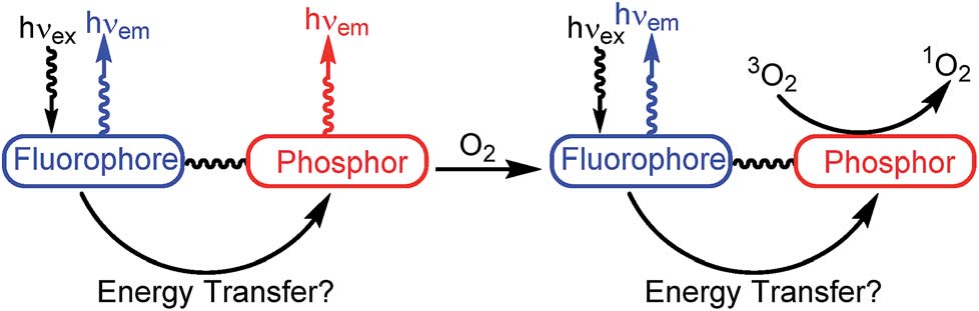
Design of ratiometric phosphor–fluorophore conjugates for sensing oxygen.

Conventional designs for ratiometric oxygen sensors partner a fluorescent molecule or nanomaterial with a molecular phosphor; in the presence of O_2_ phosphorescence is quenched whereas fluorescence is unaffected. A schematic of this sensing mechanism is summarized in Fig. 1. The diagram in Fig. 1 assumes fluorophore emission which is higher energy (bluer) than the phosphor emission, but the opposite configuration is also possible, provided energy transfer from the phosphor to fluorophore can be minimized. Excitation of the construct could then be followed by energy transfer between the components, although in many cases energy transfer from the fluorophore to the phosphor is minimal, given the short lifetime of the fluorophore. Nevertheless, if both sites can be excited simultaneously, in the absence of O_2_ fluorescence and phosphorescence from the respective sites occur. When O_2_ is added, the triplet excited state of the phosphor is quenched, reducing the phosphorescence signal. Important criteria for effective sensor design include fluorescence and phosphorescence wavelengths that are far enough apart to be resolved, and minimal energy transfer between the two components to ensure that both emission signals are observed. Along these lines, recently reported designs for ratiometric O_2_ sensors include quantum dots decorated with phosphorescent metal complexes,^12^ metal–organic frameworks with both fluorescent and phosphorescent linkers,^13^ fluorescent polymers with covalently linked cyclometalated iridium complexes,^14^ and phosphorescent cyclometalated iridium complexes tethered to coumarin fluorophores.^5^ Other relevant designs feature platinum porphyrin phosphors, either physically embedded into^15^ polymer beads along with a naphthalamide fluorophore, or covalently attached to fluorescent polymers.^16^ In addition to organometallic and organic luminophores, lanthanide ions, which emit from metal-centered excited states, have been incorporated into ratiometric luminescent oxygen sensors.^17^ Cyclometalated iridium complexes are especially attractive as the phosphorescent component, offering a combination of color tunability, high photoluminescence quantum yield, and relatively long (*ca.* μs) lifetimes not found in any other class of molecular phosphor. These attributes of cyclometalated iridium(iii) complexes have been outlined in many recent studies^18^–^27^ and have made these compounds prime candidates for a number of optoelectronic applications, including organic light emitting diodes (OLEDs)^28^–^31^ as phosphorescent chemosensors,^32^–^44^ and singlet oxygen (^1^O_2_) sensitizers.^45^–^52^

Despite their potential advantages, there are comparatively few reports using cyclometalated iridium complexes in ratiometric O_2_ sensors. One recent design which functioned well for intracellular oxygen sensing pairs a red-emitting cyclometalated iridium phosphor with a blue-emitting coumarin fluorophore, tethered to one another *via* a biologically compatible tetraproline linker.^5^ The complex linker was needed to minimize energy transfer between the components and allow simultaneous detection of both emission signals. Although this construct was ultimately effective as a ratiometric oxygen probe, synthesis of this sensor required at least 10 steps to prepare the fluorophore/linker construct, which was then coupled to the cyclometalated iridium fragment in 19% yield. There are some known constructs which combine bis-cyclometalated iridium complexes with BODIPY fluorophores,^53^–^58^ offering the advantages of BODIPY's high fluorescence quantum yields and biological compatibility,^59^–^62^ but these have not been developed as ratiometric oxygen sensors. Inspired by this previous work, here we introduce a class of ratiometric sensors prepared by a simple, modular approach, offering rapid access to several sensors with a range of emission profiles and ratiometric responses. These constructs pair bis-cyclometalated iridium fragments with BODIPY fluorophores, joined *via* variable pyridine-based linkers. The BODIPY and iridium precursors are each prepared in 1–3 steps from commercial precursors, and can be coupled to one another in yields ranging from 52% to 93%. This improved synthesis is enabled by a highly reactive cyclometalated iridium synthon recently disclosed by our group, and it is very easy to control the phosphorescence color by changing the cyclometalating ligand. Furthermore, the linker between the iridium and BODIPY influences the extent of energy transfer between the components, providing additional control over the photoluminescence output. By varying the cyclometalating ligand and linker it is possible to engender some of these compounds with dual luminescence. These dual-emitting compounds function effectively as ratiometric sensors for hypoxic environments, with high sensitivity and a large dynamic range for measuring oxygen partial pressure in abiological medium.

## Results and discussion

### Synthesis of Ir–BODIPY constructs

**Scheme 1:**
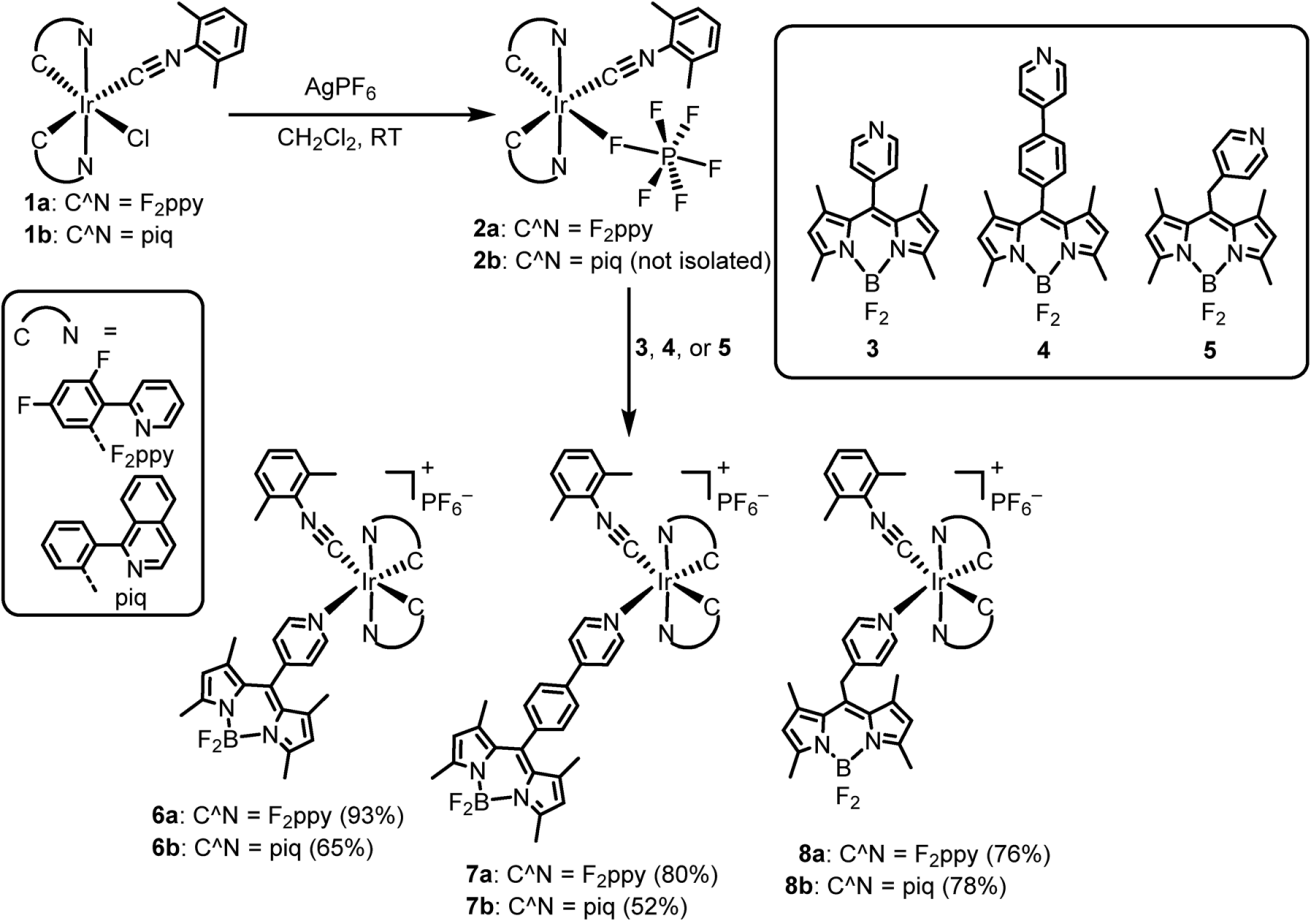
Synthesis of cyclometalated iridium–BODIPY constructs.

Scheme 1 presents the synthetic method to generate complexes with a phosphorescent [Ir(C^N)_2_]^+^ center linked to a fluorescent BODIPY chromophore. Pyridyl-substituted BODIPY precursors **3–5** with three types of linkers at the *meso*-position were prepared. Compounds **3** and **4**, with a *meso*-pyridyl and extended *meso*-4-pyridylphenyl linker, respectively, were accessed following known procedures,^63^,^64^ and the Lewis acid–base chemistry of these versions have been recently described by our group.^65^ Methylene-spaced 4-pyridinyl–CH_2_–BODIPY **5**, with an unconjugated linker between the pyridine moiety and the BODIPY core, can be prepared by the previously reported synthetic method involving a palladium-catalyzed Suzuki cross-coupling reaction.^66^ The bis-cyclometalated iridium fragment originates from precursors of the type Ir(C^N)_2_(CNAr^dmp^)(FPF_5_) (**2a** and **2b**; C^N = cyclometalating ligand, CNAr^dmp^ = 2,6-dimethylphenyl isocyanide), accessed *via* silver-mediated halide abstraction from the respective chloride-bound precursors **1a** and **1b**.^67^ For this study the two cyclometalating ligands 2-(2-,4-difluorophenyl)pyridine (F_2_ppy, **1a**/**2a**) and 1-phenylisoquinoline (piq, **1b**/**2b**) were chosen, which are known to engender the complexes with blue or red phosphorescence, respectively. Two slight variations in procedure were used to generate the reactive PF_6_^–^ precursors and combine them with the BODIPY. For the F_2_ppy complexes, the isolated precursor Ir(F_2_ppy)_2_(CNAr^dmp^)(FPF_5_) (**2a**) was treated with BODIPYs **3–5** in CH_2_Cl_2_, allowing rapid room-temperature assembly of adducts **6a–8a**, isolated in yields of 76–93%. The same procedure was unsuccessful for the preparation of the piq analogues, so instead a one-pot reaction involving Ir(piq)_2_(CNAr^dmp^)(Cl) (**1b**), AgPF_6_, and the respective BODIPY was carried out to synthesize **6b–8b**, isolated in 52–78% yield. Both procedures require silica gel column chromatography and recrystallization to separate small amounts of unreacted BODIPY and unidentified side products, following which the complexes are deemed pure by ^1^H, ^19^F, and ^11^B NMR spectra, shown in Fig. S1–S18 of the ESI.†

**Fig. 2:**
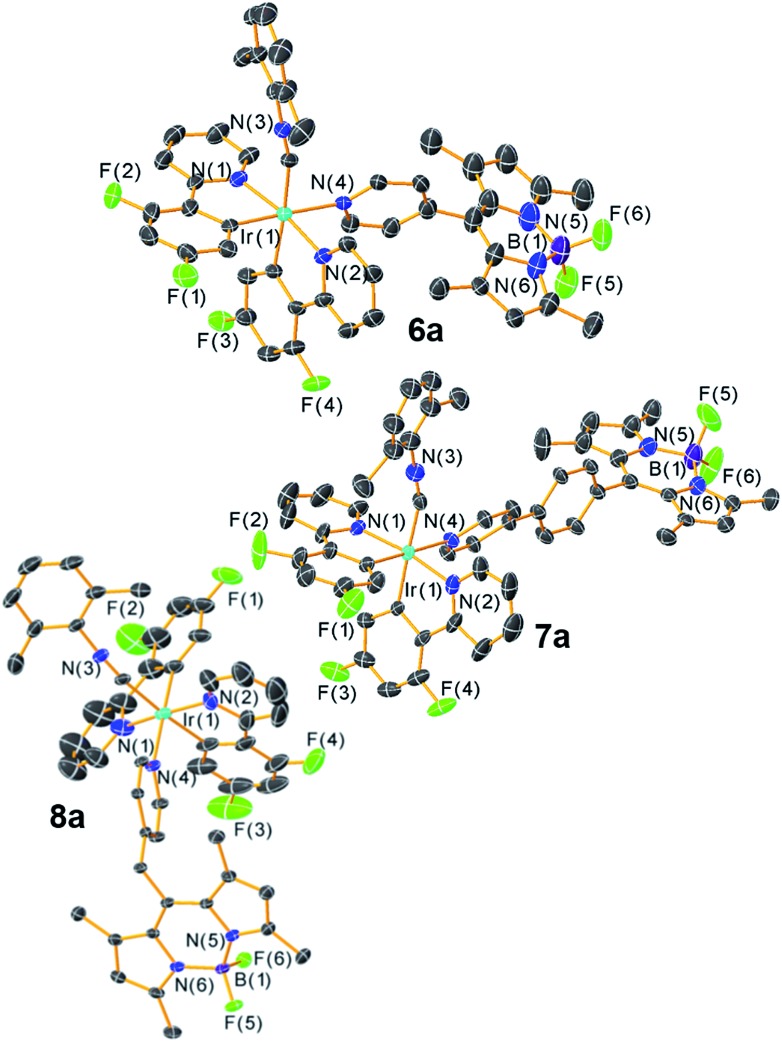
X-ray crystal structures of complex **6a–8a**. Ellipsoids are drawn at the 50% probability level with counterions, solvent molecules, and hydrogen atoms omitted.

The structures of the [Ir(F_2_ppy)(CNAr^dmp^)(BODIPY)](PF_6_) complexes **6a–8a** were ascertained by single-crystal X-ray diffraction‡
‡**6a**: CCDC 1883526, C_49_H_39_BF_12_IrN_6_P, *M* = 1173.84, monoclinic, *P*2_1_/*c*, *a* = 18.512(5) Å, *b* = 18.686(5) Å, *c* = 32.097(8) Å; *β* = 91.307(3)°, *Z* = 8, 56 882 tot. refln., 23 916 ind. refln., *R*_int_ = 0.055, *R*_1_ = 0.076, w*R*_2_ = 0.242. **7a**·1.5CH_2_Cl_2_: CCDC 1883527, C_56.50_H_46_BCl_3_F_12_IrN_6_P, *M* = 1377.32, triclinic, *P[combining macron]*1, *a* = 10.868(3) Å, *b* = 14.021(3) Å, *c* = 20.638(5) Å; *α* = 105.005(3), *β* = 92.613(3)°, *γ* = 95.539(3), *Z* = 2, 16 782 tot. refln., 12 744 ind. refln., *R*_int_ = 0.020, *R*_1_ = 0.084, w*R*_2_ = 0.311. **8a**·CH_2_Cl_2_: CCDC 1883528, C_51_H_43_BCl_2_F_12_IrN_6_P, *M* = 1272.79, monoclinic, *C*2/*c*, *a* = 45.386(6) Å, *b* = 11.4399(14) Å, *c* = 20.573(3) Å; *β* = 102.781(1)°, *Z* = 8, 54 925 tot. refln., 11 935 ind. refln., *R*_int_ = 0.025, *R*_1_ = 0.062, w*R*_2_ = 0.154. and are shown in Fig. 2. Diffraction data and refinement details for complexes **6a–8a** are summarized in Table S1 of the ESI.† The X-ray structures verify that the BODIPY attaches to the iridium center through a covalent bond with Ir–N_pyridyl_ distances ranging from 2.173(5) Å to 2.222(8) Å. The nature of the linker between the iridium and the BODIPY has measurable effects on the relative orientation of the two components. The internuclear distance between the iridium and boron atoms are similar for **6a** (9.38(1) Å, average of two independent molecules) and methylene-spaced **8a** (9.140(6) Å), whereas the additional phenyl spacer in **7a** results in a substantially longer distance of 13.73(1) Å. The alignment of the BODIPY relative to the iridium fragment also differs considerably in the three structures, as measured by the dihedral angle between the mean plane of the BODIPY and the mean plane of the F_2_ppy ligand *trans* to the BODIPY pyridyl. In complex **7a** this angle is 7.2°, indicating a nearly parallel arrangement of the BODIPY, and it increases to 21.1° in **6a** (average of two crystallographically independent molecules), and with the sp^3^ linker in **8a** the angle is 73.9°.

The identities of [Ir(C^N)_2_(CNAr^dmp^)(BODIPY)](PF_6_) complexes **6–8** were also confirmed by high resolution mass spectrometry (ESI), which clearly show the 1 : 1 combination of the bis-cyclometalated iridium fragment with the pyridyl-substituted BODIPY in each case. For each compound a strong molecular ion peak was located, the mass being a good match for the corresponding [M–PF_6_]^+^ ion. Fig. S27–S32 of the ESI† show the experimental data for the [M–PF_6_]^+^ ion juxtaposed with the simulated pattern. In each case, the isotope peaks are separated by 1 *m*/*z* unit, confirming the +1 charge of the ion, and there is a good match between the experimental and simulated isotopologue ratios. To summarize the characterization of the Ir–BODIPY constructs, the combination of X-ray crystallography and HRMS confirms the identity of the compounds, whereas multinuclear NMR validates their bulk purity.

### Photophysical properties

The UV-vis absorption and emission spectra of the free BODIPY complexes **3–5**, measured in CH_2_Cl_2_, are shown in Fig. S19 and S20 of the ESI.† To summarize briefly, the UV-vis absorption profiles are dominated by intense visible absorption with *λ*_max_ = 505 nm (*ε* = 82 000 M^–1^ cm^–1^) and *λ*_max_ = 503 nm (*ε* = 82 000 M^–1^ cm^–1^) for **3** and **4**, respectively. The absorption coefficient of methylene-spaced **5** (*ε* = 96 000 M^–1^ cm^–1^) at *λ*_max_ (506 nm) is slightly larger than that of the analogues with aromatic spacers. All of organic chromophores **3–5** are fluorescent at room temperature, and the steady-state and time-resolved emission data are summarized in Table S2 of the ESI.† All three pyridyl-BODPYS have similar photoluminescence spectra, the notable difference being the quantum yields, which for methylene-spaced **5** is observed to be 0.99, compared to 0.30 (**3**) and 0.43 (**4**) for the aromatic pyridyl-BODIPYs. This exceptionally high quantum yield for complex **5** has also been observed in other solvents such as toluene (*Φ*_PL_ = 1.0) and ethanol (*Φ*_PL_ = 0.89), as well as in other BODIPYs with substituted benzyl substituents at the *meso* position.^66^ The fluorescence lifetimes for all of the free BODIPYs are in the ns range, with a slightly longer value for **5** (6.6 ns) when compared with **3** and **4** (1.9 and 2.7 ns, respectively). The free BODIPY compounds all have nearly identical radiative rate constants (*k*_r_), so the larger quantum yield and longer lifetime in **5** is attributed to a much smaller nonradiative rate constant (*k*_nr_, see data in Table S2†). It has been established that *k*_nr_ is strongly influenced by rotation of the *meso* substituent, suggesting that the methylene-spaced substituent in **5** is more rigid than the aromatic groups in **3** and **4**.

**Fig. 3:**
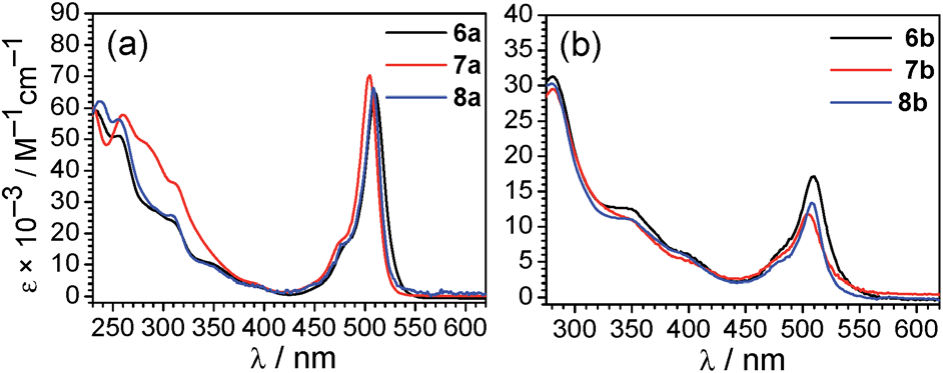
Overlaid UV-vis absorption spectra (a) of [Ir(F_2_ppy)_2_(CNAr^dmp^)(BODIPY)](PF_6_) complexes **6a–8a** and (b) of [Ir(piq)_2_(CNAr^dmp^)(BODIPY)](PF_6_) complexes **6b–8b**. Absorption spectra were recorded at room temperature in CH_2_Cl_2_.

The UV-vis absorption spectra of all Ir–BODIPY conjugates **6–8** measured at room temperature in dichloromethane are shown in Fig. 3 and summarized in Table S3 of the ESI.† The UV-vis absorption spectra of the Ir–BODIPY constructs are dominated in the visible region by strong bands in the range of 504–510 nm originating from the BODIPY. The wavelengths of these visible absorption bands are very similar to the free BODIPYs, but the molar absorptivities (*ε*) are attenuated to a significant extent, especially in the piq series. The large attenuation of *ε* in the piq series is reproducible over multiple samples, though the origin is not clear. Such an effect has not been previously observed in other 5d metal complexes linked to BODIPY through the *meso* position.^54^,^68^–^72^ The UV and near-visible regions involve several overlapping bands, attributed to a combination of π → π* transitions involving the BODIPY (compare to Fig. S19†), and the expected bands^73^ from the [Ir(C^N)_2_]^+^ fragment that include C^N-centered π → π* transitions at higher energy and MLCT transitions at lower energy.

**Fig. 4:**
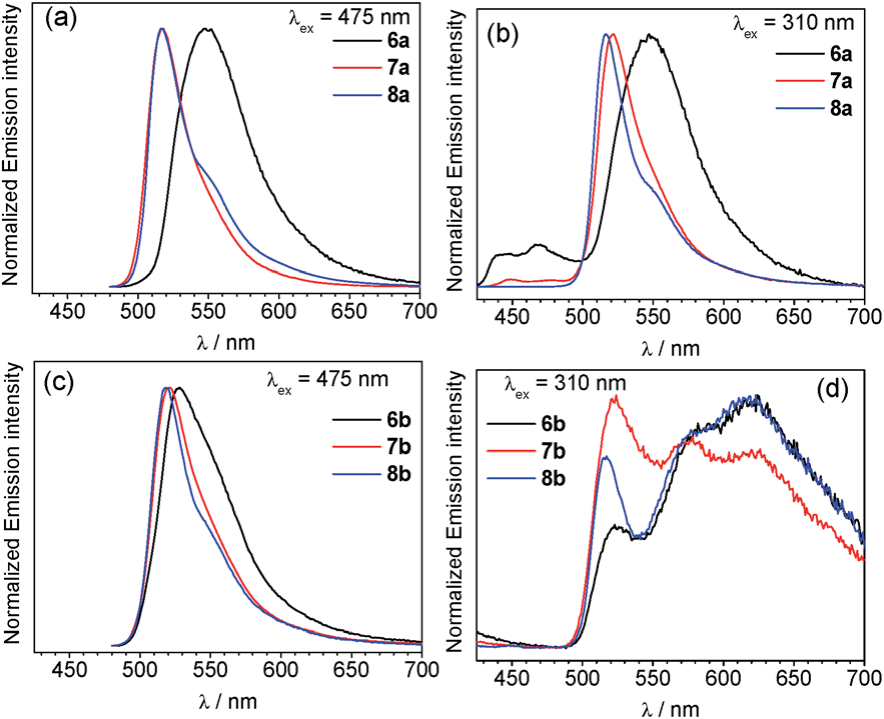
Photoluminescence spectra (a) F_2_ppy complexes **6a–8a**, excited at 475 nm, (b) **6a–8a** excited at 310 nm, (c) piq complexes **6b–8b** excited at 475 nm, and (d) **6b–8b** excited at 310 nm. All spectra were recorded at room temperature in deoxygenated CH_2_Cl_2_.

Overlaid photoluminescence spectra of complexes **6–8** are displayed in Fig. 4, and the data is summarized in Table 1. The spectra are excitation-wavelength dependent, and with 475 nm excitation, where only the BODIPY is expected to absorb, only fluorescence coming from the BODIPY fragment is observed. In most cases the fluorescence wavelength is nearly identical to the respective free BODIPY. However in complex **6a**, where the [Ir(F_2_ppy)_2_(CNAr^dmp^)]^+^ fragment is linked to the BODIPY *via* a short pyridyl spacer, the BODIPY fluorescence is significantly red-shifted to 549 nm, and while not as pronounced the fluorescence in the piq complex with the same linker (**6b**) red-shifts to 528 nm. This measurable red shift of the fluorescence upon coordination to iridium is similar to the effect we observed previously when Lewis acidic boranes were coordinated to BODIPY **3**.^65^ Lifetimes for the fluorescence component are quite similar to the free BODIPYs **3–5** (Table S2†). In most cases the fluorescence quantum yields, determined when the BODIPY is selectively excited at 475 nm, are significantly lower than the free BODIPY and range from 7.4–66% across the series of compounds.

**Table 1:** Summary of photophysical properties of Ir–BODIPY complexes **6–8**. Emission spectra were measured in CH_2_Cl_2_ at 293 K

	*λ*_em_/nm^*a*^	*λ*_em_/nm^*b*^	*Φ*_PL_ ^*a*^	*Φ*_PL_ ^*b*^	*τ*/ns^*d*^	*τ*/μs^*e*^
**6a**	549	438, 465, 550	0.074	0.060	1.4	6.8
**7a**	517	448, 472(sh), 521	0.30	0.13	2.7	5.6
**8a**	516	517^*c*^	0.66	0.65	5.2	N.D.^*c*^
**6b**	528	527, 585(sh), 621	0.089	0.041	2.5	6.0
**7b**	521	520, 576, 620	0.16	0.063	1.5	6.9
**8b**	518	515, 574, 619	0.13	0.052	5.2	7.3

^*a*^Fluorescence only, excited at 475 nm.

^*b*^Fluorescence and phosphorescence excited at 310 nm.

^*c*^No phosphorescence for this compound.

^*d*^Fluorescence lifetimes, excited at 455 nm.

^*e*^Phosphorescence lifetime, excited at 330 nm.

In contrast, with 310 nm excitation, where both the iridium phosphor and the BODIPY fluorophore absorb, two emission bands are observed in most cases. Iridium-centered phosphorescence, with wavelengths that depend on the identity of the cyclometalating ligand, is observed in addition to the BODIPY-centered fluorescence. This vibronically structured band occurs with *λ*_max_ at *ca.* 440 and 467 nm in F_2_ppy complexes **6a** and **7a**, and *λ*_max_ at *ca.* 575 and 623 nm for piq complexes **6b–8b**. No phosphorescence is observed in complex **8a**, where C^N = F_2_ppy and the BODIPY includes a methylene spacer, suggesting that in this case there is efficient energy transfer from the [Ir(F_2_ppy)_2_]^2+^ fragment to the BODIPY, and only BODIPY-centered fluorescence is observed at either excitation wavelength. The more efficient energy transfer in **8a** seems to indicate that energy transfer does not involve electronic coupling through the *meso* position, which is expected to be minimal in **8a** given the unconjugated linker. Thus the slight differences in energy transfer in **6a–8a** are likely a function of the different spatial arrangements of the BODIPY acceptor relative to the iridium donor, as observed crystallographically in the solid state (see above). Energy transfer from BODIPY to Ir does not appear to occur, since the red phosphorescence in piq complexes **6b–8b** is only observed when the Ir center is excited directly, and does not occur when only the BODIPY is excited at 475 nm. The photoluminescence quantum yields (*Φ*_PL_) of the Ir–BODIPY conjugates tend to be somewhat lower when both the Ir and the BODIPY are simultaneously excited at 310 nm, ranging between 6.0 and 65%. The one exception is complex **8a**, which only exhibits fluorescence at both excitation wavelengths and has the same quantum yield in each case, again consistent with efficient energy transfer from Ir to BODIPY for this compound. For the rest of the compounds, the wavelength-dependence of the quantum yield suggests that phosphorescence from the iridium center is inherently less efficient than fluorescence from the BODIPY. In **6a** and **7a** it is difficult to evaluate whether excited-state energy transfer between the [Ir(F_2_ppy)_2_]^2+^ center and the BODIPY is occurring, though we presume there is some degree of energy transfer since the phosphorescence quantum yield in these compounds is much lower than we typically observe for isocyanide-bound [Ir(F_2_ppy)_2_]^+^ complexes.^73^,^74^ The phosphorescence lifetimes of all complexes (except **8a**, which doesn't phosphoresce) fall in the narrow range of 5.6–7.3 μs.

The apparent dual emission with UV excitation persists and is identical in samples of the Ir–BODIPY complexes that have been purified multiple times by silica gel column chromatography and recrystallization, suggesting it is not a result of minor impurities. In addition, the excitation spectra for **6–8** (Fig. S21–S26†) overlay very well with the UV-vis absorption, indicating that both emission bands arise from the Ir–BODIPY conjugate. These investigations suggest that the orientation of the BODIPY chromophore relative to the iridium center, controlled by the nature of the linker between the fluorophore component and phosphor fragment, can play a role in the energy-transfer dynamics and photoluminescence properties. In general when C^N = F_2_ppy energy transfer can occur such that predominantly BODIPY fluorescence is observed, whereas when C^N = piq there is minimal energy transfer and dual emission occurs when both sites are excited. The relative amounts of phosphorescence and fluorescence do vary somewhat across the series as a function of the linker, although the ratio of phosphorescence to fluorescence is also excitation-wavelength dependent.

### Ratiometric oxygen sensing

Whereas complexes **7a** and **8a** have very weak or negligible phosphorescence and are not good candidates for ratiometric oxygen sensing, the remaining four compounds have comparable levels of phosphorescence and fluorescence and were evaluated as ratiometric O_2_ sensors. To qualitatively investigate their response to O_2_, samples photoluminescence spectra were collected in deoxygenated solutions, prepared in a nitrogen-filled glovebox, and compared to spectra for air-equilibrated samples. As shown in Fig. S33,† after air exposure the signal arising from iridium-centered phosphorescence, which occurs in the blue region for **6a** and in the red for **6b–8b**, disappears completely. The fluorescence signal from the BODIPY is minimally altered.

**Fig. 5:**
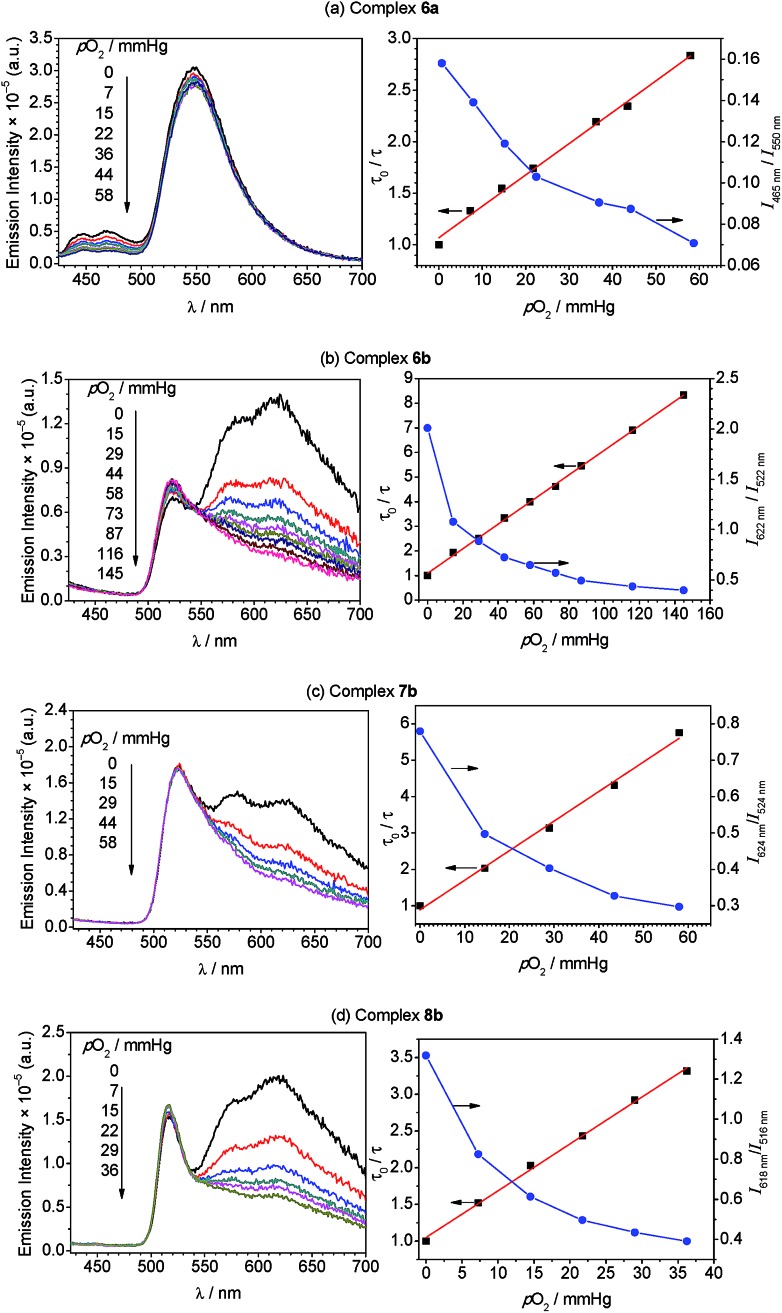
Oxygen sensing data for complexes **6a** (a), **6b** (b), **7b** (c), and **8b** (d). The left-hand plots show photoluminescence spectra (*λ*_ex_ = 310 nm) of the respective Ir–BODIPY complex (5 μM) measured in CH_2_Cl_2_ at room temperature under various oxygen partial pressures. The right-hand plots overlay the ratiometric response (blue circles) and Stern–Volmer plot (black squares, with linear fit) as a function of oxygen partial pressure. For the ratiometric data, the solid line is drawn merely as a guide, and the ratio was determined from integrated emission intensities below 500 nm (phosphorescence) and above 500 nm (fluorescence) for complex **6a**, and for emission signal at the peak wavelengths for complexes **6b–8b**.

To more quantitatively evaluate the sensing response, photoluminescence measurements were carried out at varying *p*O_2_ levels, until the point when the phosphorescence signal no longer changed appreciably. These results are summarized in Fig. 5, which shows the spectral evolution for each compound as *p*O_2_ is gradually increased. All four of the complexes are able to sense low levels of oxygen below atmospheric content (*p*O_2_ ≤ 160 mmHg), indicating they are suitable for hypoxic environments. The right-hand plots of Fig. 5 (blue circles) show the sensing response plotted as the ratio of phosphorescence to fluorescence signal, determined from the intensities at the respective maxima, *vs. p*O_2_. These plots demonstrate that the ratiometric response spans a >3-fold range in each case, *i.e.* the signal ratio changes by more than a factor of three over the range of *p*O_2_ values tested.

To gain further insight into the oxygen sensing response, Stern–Volmer analysis on the phosphorescence lifetime was conducted. Eqn (1) shows the Stern–Volmer relationship,^75^,^76^ where *K*_sv_ is the Stern–Volmer constant, *p*O_2_ is the oxygen partial pressure, *k*_q_ is the quenching rate constant, and *τ*_0_ and *τ* are the lifetimes in the absence and presence of oxygen, respectively.1
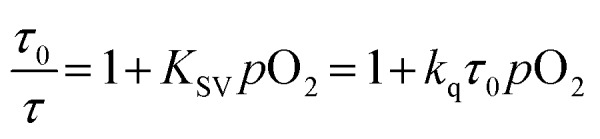



The Stern–Volmer plots and linear fits are overlaid with the ratiometric sensing data in in Fig. 5, and the Stern–Volmer parameters extracted from this data are summarized in Table 2. The Stern–Volmer constant *K*_sv_ can be obtained from the slope of the linear fit. Also, the quenching rate constant *k*_q_ can be determined by using *K*_sv_ and *τ*_0_. The Stern–Volmer constants (*K*_sv_) for bis-cyclometalated iridium–BODIPY complexes **6a**, and **6b–8b** were determined to be 3.0–8.1 × 10^–2^ mmHg^–1^, and from these values quenching rate constants (*k*_q_) were determined to vary from 4.4 × 10^3^ s^–1^ mmHg^–1^ to 1.2 × 10^4^ s^–1^ mmHg^–1^. To convert the unit of the quenching rate constant to s^–1^ M^–1^, which is more conventional for bimolecular quenching rate constants, we adopted the previously reported literature^77^ regarding to the solubility of oxygen into organic solvents. The solubility value of oxygen into dichloromethane at 298.2 K and 101.33 kPa (1 atm) is 7.09 × 10^–4^ M, and using this conversion factor *k*_q_ ranges from 4.7 × 10^9^ s^–1^ M^–1^ to 1.3 × 10^10^ s^–1^ M^–1^ in the four Ir–BODIPY conjugates evaluated as sensors. To contextualize these results, we see that all four sensors presented here are efficiently quenched by O_2_, with *k*_q_ values approaching the diffusion limit. The Stern–Volmer kinetics seem to depend slightly on the C^N ligand, with the complexes in the piq series (**6b–8b**) quenching more efficiently than F_2_ppy complex **6a**. For **6b–8b** there are slight differences in Stern–Volmer parameters, indicating there may be a small dependence on the nature of the BODIPY spacer, but all three behave quite similarly overall. Comparing to previously described ratiometric oxygen sensors, the *K*_SV_ values for the Ir–BODIPY constructs described here are larger than those of a recently described MOF-based nanosensor^13^ and a platinum porphyrin polymer sensor,^16^ both of which have *K*_sv_ = 1.7 × 10^–2^ mmHg^–1^, and comparable to a cyclometalated iridium-coumarin sensor with *K*_sv_ = 6.4 × 10^–2^ mmHg^–1^.^5^ Comparing to sensors with reported quenching rate constants, the complexes described here have *k*_q_ values that exceed those of a nanocrystal sensor decorated with osmium phosphors (*k*_q_ = 1.8 × 10^9^ s^–1^ M^–1^)^12^ and protoporphyrin IX (*k*_q_ = 4.0 × 10^2^ s^–1^ mmHg^–1^),^78^ which functions as an endogenous turn-off O_2_ sensor. These comparisons demonstrate that the new ratiometric oxygen sensors described here compare favorably with well-known examples of biological oxygen sensors, with good sensitivity (*k*_q_ up to 1.3 × 10^10^ s^–1^ M^–1^) and dynamic ranges suitable for hypoxic measurements (<160 mmHg).^79^,^80^

**Table 2:** Stern–Volmer constants (*K*_sv_) and the quenching rate constants (*k*_q_) for complexes **6a** and **6b–8b**

Entry	*K*_sv_/mmHg^–1^	*k*_q_/s^–1^ mmHg^–1^	*k*_q_/s^–1^ M^–1^
**6a**	3.0 × 10^–2^	4.4 × 10^3^	4.7 × 10^9^
**6b**	5.0 × 10^–2^	8.3 × 10^3^	8.9 × 10^9^
**7b**	8.1 × 10^–2^	1.2 × 10^4^	1.3 × 10^10^
**8b**	6.4 × 10^–2^	8.8 × 10^3^	9.4 × 10^9^

## Conclusions

In summary, we have developed two-component assemblies with a phosphorescent metal center ([Ir(C^N)_2_(CNAr^dmp^)]^+^) linked to the well-known fluorophore BODIPY and demonstrated their utility as ratiometric O_2_ sensors. The constructs are very simple to prepare in high yields using a modular approach where the synthetic linchpin is a labile cyclometalated iridium precursor with a loosely bound PF_6_^–^ anion. These Ir–BODIPY conjugates exhibit dual luminescence where fluorescence originates from the organic chromophore and phosphorescence comes from the iridium center, and the spectral profile is dependent on the identity of the C^N ligand as well as the nature of the linker between the iridium center and the BODIPY. Complexes with C^N = F_2_ppy in general do not function well as sensors, since energy transfer from the long-lived iridium-centered excited state to the BODIPY is favorable and efficient, resulting in very weak phosphorescence signal. In contrast, for complexes with C^N = piq, where the triplet excited state is lower in energy than the BODIPY singlet state, energy transfer is shut down and comparable amounts of luminescence from both sites are observed, the precise ratio varying slightly with the nature of the linker between BODIPY and iridium. Evaluation of the oxygen-dependent photoluminescence, in concert with Stern–Volmer quenching analysis of the phosphorescence lifetime, demonstrates that four compounds described here function as effective ratiometric O_2_ sensors with sensitivities and dynamic ranges suitable for hypoxic environments. This conceptually simple design, where the fluorophore and phosphor are linked *via* a modular and simple synthetic strategy, should be applicable to a range of next-generation oxygen sensors. Specific future improvements include the design of sensors with greater brightness (higher quantum yields) and larger spectral separation between the fluorescence and phosphorescence signal, both of which should improve sensitivity and dynamic range.

## Experimental section

General synthetic procedures and key details of physical measurements are included here. Full experimental details are provided in the ESI.†

### Synthesis of Ir–BODIPY complexes

#### General synthetic procedure for [Ir(F_2_ppy)_2_(CNAr^dmp^)–(BODIPY)](PF_6_) complexes **6a–8a**

A mixture of Ir(F_2_ppy)_2_(CNAr^dmp^)(FPF_5_) (**1a**) (1 equiv.) and the respective BODIPY compound **3–5** (1 equiv.) was dissolved in 10 mL of CH_2_Cl_2_ in a vial in the glovebox. The solution was stirred at room temperature for 18 h. The solvent was removed under reduced pressure and the resulting solid was washed three times with hexane. The residue was purified by silica gel column chromatography (SiO_2_) eluting with ethyl acetate/CH_2_Cl_2_ (1 : 4 v/v) to get the desired product, which was further purified by recrystallization (dichloromethane/hexane).

#### General procedure to prepare [Ir(piq)_2_(CNAr^dmp^)(BODIPY)](PF_6_) complexes **6b–8b**

Ir(piq)_2_(CNAr^dmp^)(Cl) (**1b**) (1 equiv.) and BODIPY compound **3–5** (1 equiv.) were dissolved in 15 mL of CH_2_Cl_2_ in a 25 mL round-bottom flask in the glovebox and stirred at room temperature for 1 h. AgPF_6_ (1 equiv.) was added to the orange mixture which was stirred for 24 h at room temperature. The AgCl was filtered off and the solvent was removed under vacuum. The orange solid was washed three times with hexane. The residue was purified by column chromatography, using a silica gel (SiO_2_) and 1 : 4 (v/v) ethyl acetate/CH_2_Cl_2_ as the eluent, followed by recrystallization (dichloromethane/hexane).

#### Oxygen sensing and Stern–Volmer experiments

Dichloromethane solutions of Ir–BODIPY conjugates **6a** and **6b–8b** were prepared in a nitrogen-filled glovebox. These stock solutions were diluted in a quartz cuvette to concentrations of 5.0–7.0 × 10^–6^ M. Using a microliter syringe, 50–100 μL aliquots of air were introduced to the cuvette. The lifetimes and emission spectra were measured in the nitrogen atmosphere (*p*O_2_ = 0 mmHg) and following addition of each aliquot of air, up to atmospheric conditions (*p*O_2_ = 160 mmHg). For emission spectra, the samples were excited at 310 nm, and for lifetime decay, 330 nm excitation was used. Using the changes of lifetimes in various oxygen partial pressures, the Stern–Volmer relationship was used to extract Stern–Volmer quenching constants (*K*_sv_) and the quenching rate constant (*k*_q_).

## Conflicts of interest

There are no conflicts to declare.

## Supplementary Material

Supplementary information

Crystal structure data
