# Supplementary material for: Cyclometalated iridium–BODIPY ratiometric O2 sensors
Source: Chem Sci. 2019 Apr 15;10(19):5124–32. doi: 10.1039/c9sc00696f (PMC6524664; doi:10.1039/c9sc00696f)
Supplement: Supplementary file 1 [file SC-010-C9SC00696F-s001.pdf]

Electronic Supplementary Material (ESI) for Chemical Science.
This journal is © The Royal Society of Chemistry 2019

Supplementary Material (ESI) for

Cyclometalated Iridium-BODIPY Ratiometric O_2 Sensors

Ku Sun Choung, Karen Marroquin, and Thomas S. Teets*

Department of Chemistry, University of Houston,
3585 Cullen Blvd. Room 112, Houston, TX 77204-5003, USA
email: tteets@uh.edu

Index	*Page*
Experimental Section	S2–S4
X-ray crystallography summary table	S5
NMR spectra of all new complexes	S6–S23
UV-vis absorption and emission spectra of BODIPYs **3**–**5**	S24
Summary of UV-vis absorption and emission data	S25
UV-vis absorption and excitation spectra of complexes **6**–**8**	S26–S31
High resolution mass spectrometric analysis of complexes **5**–**10**	S32–S37
Emission spectra of complexes **6a** and **6b**–**8b** under inert and aerobic atmospheres.	S38
References	S39

Experimental Section

Materials. Dry solvents were obtained from a Grubbs Solvent Purification System and deaerated with argon. Starting materials and reagents, unless otherwise specified, were obtained from commercial sources and used without further purification. 4-pyridinyl BODIPY complex **3** was prepared by the published method using 2,4-dimethylpyrrole, pyridine-4-carboxaldehyde, and chloranil.[1,2] 4-phenyl-4-pyridinyl BODIPY complex **4** was prepared by the synthetic procedure previously reported by our group.[3] 4-Pyridinyl-CH_2-BODIPY complex **5** was prepared according to the published synthetic procedure using a palladium-catalyzed Suzuki cross-coupling reaction.[4] The starting materials Ir(C^N)$_2$(CNAr^{dmp})(Cl) (**1a**: C^N = F_2ppy; **1b**: C^N = piq) and Ir(F_2ppy)$_2$(CNAr^{dmp})(FPF$_5$) (**2a**) were prepared as previously described by our group.[5]

Physical Methods. ^{1}H, ^{13}C, ^{19}F, and ^{11}B NMR spectra were recorded at room temperature using a JEOL ECA-400 or ECA-500 spectrometer. UV-vis absorption spectra were recorded in screw-capped 1 cm quartz cuvettes using an Agilent Carey 8454 UV-vis spectrophotometer. Steady-state emission spectra were recorded using a Horiba FluoroMax-4 spectrofluorometer. To exclude air, samples for emission spectra were prepared in a nitrogen-filled glove-box using dry, deoxygenated toluene. Emission quantum yields were obtained by a relative method using 4-Pyridinyl BODIPY **3** as the standard(Φ_F = 0.30, λ_{ex} = 310 or 475 nm).[1] The emission spectra of the Ir-BODIPY complexes and the standard were measured with a range of absorbance between 0.01 and 0.1 at the excitation wavelengths mentioned above. The integrated emission intensity was plotted vs. absorbance and the slope of the best-fit line was obtained. The quantum yield of the Ir-BODIPY conjugates (Φ_x) was calculated using Equation 1 below, where Φ_{st} = the quantum yield of the standard, m_x = the slope for the samples, m_{st} = standard compound, η_x and η_{st} are the refractive indexes of the solvents of the sample and standard, respectively).

$$\Phi_x = \Phi_{st}\left[\frac{m_x}{m_{st}}\right]\left[\frac{\eta_x}{\eta_{st}}\right]^2 \qquad (1)$$

Luminescence lifetimes were measured with a Horiba DeltaFlex Lifetime System, using pulsed diode excitation. Emission wavelengths were selected by using appropriate long-pass filters, and the decay trace was fit using the instrument's analysis software. The high-resolution mass spectrometry (HRMS-ESI) measurements were carried out by the Mass Spectrometry Laboratory at University of Houston, using a Thermo Exactive mass spectrometer and operated in positive ionization mode with a spray voltage at 1.5 kV. ^{1}H and ^{19}F NMR spectra of all new compounds are shown in the Supporting Information, Figs. S3–S20, and provide additional evidence for sample purity.

[Ir(F_2ppy)$_2$(CNAr^{dmp})(4-pyridinyl-BODIPY)](PF_6) (6a). Prepared by the general method using Ir(F_2ppy)$_2$(CNAr^{dmp})(FPF$_5$) (**2a**) (54 mg, 0.063 mmol) and 4-pyridinyl-BODIPY (**3**) (20 mg, 0.063 mmol). Yield: 69 mg (93%). ^{1}H NMR (500 MHz, $CDCl_3$) δ: 9.38 (d, J = 5.5 Hz, 1H, Ar*H*), 8.86 (br, s, 2H, Pyridine *H*), 8.41 (dd, J = 21.7, 7.7 Hz, 2H, Ar*H*), 8.22 (d, J = 8.3 Hz, 1H, Ar*H*), 8.04 (t, J = 8.5 Hz, 1H, Ar*H*), 7.93 (t, J = 7.4 Hz, 1H, Ar*H*), 7.51–7.39 (m, 4H, Ar*H*), 7.13 (d, J = 15.3 Hz, 1H, Ar*H*), 7.00 (d, J = 6.4 Hz, 2H, Pyridine *H*), 6.53 (q, J = 11.7 Hz, 2H, Ar*H*), 5.95 (s, 2H, Pyrrole *H*), 5.82 (d, J = 10.4 Hz, 1H, Ar*H*), 5.74–5.63 (m, 1H, Ar*H*), 2.49 (s, 6H, CH_3), 2.10 (s, 6H, CH_3), 1.16 (s, 6H, CH_3). ^{19}F NMR (470 MHz, $CDCl_3$) δ: −72.32 (d, J = 713.0 Hz, 6F, PF_6), −103.49 to −104.01 (m, 1F, F_2ppy), −105.11 (q, J = 9.0 Hz, 1F, F_2ppy), −107.14 (t, J = 11.7 Hz, 1F, F_2ppy), −108.38 (t, J = 12.5 Hz, 1F, F_2ppy), −145.75 to −146.02 (m, 2F, BF_2). ^{11}B NMR (160 MHz, $CDCl_3$) δ: −0.38 (t, J = 31.2 Hz, 1B, BF_2). HRMS (ESI): *m/z* calcd. for $C_{49}H_{39}BF_{12}IrN_6P$ $[M - PF_6]^+$: 1029.2863, found: 1029.2996.

[Ir(F_2ppy)$_2$(CNAr^{dmp})(4-phenyl-4-pyridinylBODIPY)](PF_6) (7a). Prepared by the general method using Ir(F_2ppy)$_2$(CNAr^{dmp})(FPF_5) (**2a**) (54 mg, 0.063 mmol) and 4-phenyl-4-pyridinyl-BODIPY (**4**) (25 mg, 0.063 mmol). Yield: 62 mg (79%). ^{1}H NMR (500 MHz, $CDCl_3$) *δ*: 9.41 (d, *J* = 1.4 Hz, 1H, Ar*H*), 8.74 (br, s, 2H, Pyridine *H*), 8.42 (d, *J* = 9.0 Hz, 1H, Ar*H*), 8.34 (d, *J* = 5.9 Hz, 1H, Ar*H*), 8.28 (d, *J* = 10.0 Hz, 1H, Ar*H*), 8.03 (t, *J* = 7.8 Hz, 1H, Ar*H*), 7.96 (t, *J* = 8.1 Hz, 1H, Ar*H*), 7.85 (d, *J* = 8.5 Hz, 2H, Ph*H*), 7.82 (s, 2H, Ar*H*), 7.51 (t, *J* = 7.4 Hz, 1H, Ar*H*), 7.42 (d, *J* = 7.4 Hz, 1H, Ar*H*), 7.39 (d, *J* = 8.4 Hz, 2H, Ph*H*), 7.19–7.13 (m, 1H, Ar*H*), 7.04 (d, *J* = 7.8 Hz, 2H, Pyridine *H*), 6.53 (dd, *J* = 12.4, 7.7 Hz, 2H, Ar*H*), 5.95 (s, 2H, Pyrrole *H*), 5.78 (d, *J* = 8.1 Hz, 1H, Ar*H*), 5.70 (d, *J* = 7.7 Hz, 1H, Ar*H*), 2.53 (s, 6H, CH_3), 2.13 (s, 6H, CH_3), 1.33 (s, 6H, CH_3). ^{19}F NMR (470 MHz, $CDCl_3$) δ: −72.64 (d, *J* = 712.6 Hz, 6F, PF_6), −104.08 to −104.40 (m, 1F, F_2ppy), −105.23 (q, *J* = 8.7 Hz, 1F, F_2ppy), −107.17 (t, *J* = 11.7 Hz, 1F, F_2ppy), −108.48 (d, *J* = 11.9 Hz, 1F, F_2ppy), −146.02 (dd, *J* = 65.7, 31.7 Hz, 2F, BF_2). ^{11}B NMR (160 MHz, $CDCl_3$) δ: −0.21 (t, *J* = 31.8 Hz, 1B, BF_2). HRMS (ESI): *m*/*z* calcd. for $C_{55}H_{43}BF_{12}IrN_6P$ [M − PF_6]$^+$: 1105.3176, found: 1105.3590.

[Ir(F_2ppy)$_2$(CNAr^{dmp})(4-pyridinyl-CH_2-BODIPY)](PF_6) (8a). Prepared by the general method using Ir(F_2ppy)$_2$(CNAr^{dmp})(FPF_5) (**2a**) (54 mg, 0.063 mmol) and 4-phenyl-CH_2-BODIPY (**5**) (21 mg, 0.063 mmol). Yield: 57 mg (76%). ^{1}H NMR (400 MHz, $CDCl_3$) δ: 9.27 (d, *J* = 5.6 Hz, 1H, Ar*H*), 8.53 (br, s, 2H, Pyridine *H*), 8.39 (d, *J* = 8.9 Hz, 1H, Ar*H*), 8.29 (d, *J* = 9.6 Hz, 1H, Ar*H*), 8.20 (d, *J* = 5.7 Hz, 1H, Ar*H*), 8.03 – 7.93 (m, 2H, Ar*H*), 7.43 (t, *J* = 6.6 Hz, 2H, Ar*H*), 7.37 (s, 2H, Ar*H*), 7.17 (t, *J* = 7.6 Hz, 1H, Ar*H*), 7.04 (d, *J* = 7.6 Hz, 2H, Pyridine *H*), 6.50 (q, *J* = 9.7 Hz, 2H, Ar*H*), 6.01 (d, *J* = 16.7 Hz, 2H, Pyrrole *H*), 5.69 (d, *J* = 10.0 Hz, 1H, Ar*H*), 5.60 (d, *J* = 9.5 Hz, 1H, Ar*H*), 4.43 (s, 2H, CH_2), 2.49 (s, 6H, CH_3), 2.18 (s, 3H, CH_3), 2.10 (s, 9H, CH_3). ^{19}F NMR (376 MHz, $CDCl_3$) δ: −72.62 (d, *J* = 712.7 Hz, 6F, PF_6), −103.91 (q, *J* = 9.5, 8.4 Hz, 1F, F_2ppy), −105.35 (q, *J* = 9.0 Hz, 1F, F_2ppy), −106.95 (t, *J* = 11.8 Hz, 1F, F_2ppy), −108.49 (t, *J* = 11.6 Hz, 1F, F_2ppy), −145.76 to −146.82 (m, 2F, BF_2). ^{11}B NMR (160 MHz, $CDCl_3$) δ: −0.41 (t, *J* = 34.4 Hz, 1B, BF_2). HRMS (ESI): *m*/*z* calcd. for $C_{50}H_{41}BF_{12}IrN_6P$ [M − PF_6]$^+$: 1043.3019, found: 1043.3406.

[Ir(piq)$_2$(CNAr^{dmp})(4-pyridinyl-BODIPY)](PF_6) (6b). Prepared by the general procedure using Ir(piq)$_2$(CNAr^{dmp})(Cl) (**1b**) (40 mg, 0.052 mmol), 4-pyridinyl-BODIPY (**3**) (17 mg, 0.052 mmol), and AgPF_6 (14 mg, 0.052 mmol). Yield: 39 mg (65%). ^{1}H NMR (500 MHz, $CDCl_3$) *δ*: 9.24 (d, *J* = 6.4 Hz, 1H, Ar*H*), 8.98 (d, *J* = 8.4 Hz, 1H, Ar*H*), 8.88 (br, s, 2H, Pyridine *H*), 8.78 (d, *J* = 8.6 Hz, 1H, Ar*H*), 8.25 (dd, *J* = 16.0, 7.2 Hz, 2H, Ar*H*), 8.15 (d, *J* = 8.0 Hz, 1H, Ar*H*), 8.09 (d, *J* = 8.0 Hz, 1H, Ar*H*), 8.04 (d, *J* = 8.2 Hz, 1H, Ar*H*), 7.90–7.81 (m, 4H, Ar*H*), 7.75 (d, *J* = 6.6 Hz, 2H, Ar*H*), 7.35 (d, *J* = 6.0 Hz, 2H, Pyridine *H*), 7.09 (dt, *J* = 12.5, 6.8 Hz, 3H, Ar*H*), 6.96 (d, *J* = 7.6 Hz, 3H, Ar*H*), 6.84 (t, *J* = 7.4 Hz, 1H, Ar*H*), 6.51 (d, *J* = 7.6 Hz, 1H, Ar*H*), 6.24 (d, *J* = 7.4 Hz, 1H, Ar*H*), 5.90 (s, 2H, Pyrrole *H*), 2.46 (s, 6H, CH_3), 2.05 (s, 6H, CH_3), 1.04 (s, 6H, CH_3). ^{19}F NMR (470 MHz, $CDCl_3$) δ: −72.69 (d, *J* = 712.7 Hz, 6F, PF_6), −145.93 (dd, *J* = 64.4, 30.8 Hz, 2F, BF_2). ^{11}B NMR (160 MHz, $CDCl_3$) δ: −0.43 (t, *J* = 32.5 Hz, 1B, BF_2). HRMS (ESI): *m*/*z* calcd. for $C_{57}H_{47}BF_8IrN_6P$ [M − PF_6]$^+$: 1057.3553, found: 1057.3947.

[Ir(piq)$_2$(CNAr^{dmp})(4-phenyl-4-pyridinyl-BODIPY)](PF_6) (7b). Prepared by the general procedure using Ir(piq)$_2$(CNAr^{dmp})(Cl) (**1b**) (40 mg, 0.052 mmol), 4-phenyl-4-pyridinyl-BODIPY (**4**) (21 mg, 0.052 mmol), and AgPF_6 (14 mg, 0.052 mmol). Yield: 35 mg (52%). ^{1}H NMR (500 MHz, $CDCl_3$) *δ*: 9.29 (d, *J* = 6.4 Hz, 1H, Ar*H*), 8.96 (d, *J* = 8.5 Hz, 1H, Ar*H*), 8.82 (d, *J* = 8.6 Hz, 1H, Ar*H*), 8.78 (br, s, 2H, Pyridine *H*), 8.23 (d, *J* = 6.3 Hz, 2H, Ph*H*), 8.15 (d, *J* = 8.0 Hz, 1H, Ar*H*), 8.07 (d, *J* = 8.1 Hz, 1H, Ar*H*), 8.03 (d, *J* = 8.1 Hz, 1H, Ar*H*), 7.83 (td, *J* = 16.8, 16.3, 7.1 Hz, 7H, Ar*H*), 7.76 (d, *J* = 5.6 Hz, 2H, Ph*H*), 7.69 (d, *J* = 6.4 Hz, 1H, Ar*H*), 7.37 (d, *J* = 8.0 Hz,

2H, Pyridine *H*), 7.14–7.09 (m, 2H), 7.07–7.02 (m, 1H, Ar*H*), 6.97 (dd, *J* = 13.8, 7.4 Hz, 3H, Ar*H*), 6.82 (t, *J* = 7.4 Hz, 1H, Ar*H*), 6.36 (d, *J* = 7.6 Hz, 1H, Ar*H*), 6.22 (d, *J* = 7.4 Hz, 1H, Ar*H*), 5.94 (s, 2H, Pyrrole *H*), 2.53 (s, 6H, C*H*3), 2.08 (s, 6H, C*H*3), 1.32 (s, 6H, C*H*3). ^{19}F NMR (470 MHz, $CDCl_3$) δ: −73.04 (d, J = 712.6 Hz, 6F, PF_6), −146.07 (dd, J = 65.8, 31.7 Hz, 2F, BF_2). ^{11}B NMR (160 MHz, $CDCl_3$) δ: −0.21 (t, *J* = 30.2 Hz, 1B, BF_2). HRMS (ESI): *m/z* calcd. for $C_{63}H_{51}BF_8IrN_6P$ $[M - PF_6]^+$: 1133.3866, found: 1133.4300.

[Ir(piq)$_2$(CNAr^{dmp})(4-pyridinyl-CH$_2$-BODIPY)](PF$_6$) (8b). Prepared by the general procedure using Ir(piq)$_2$(CNAr^{dmp})(Cl) (**1b**) (33 mg, 0.041 mmol), 4-pyridinyl-CH_2-BODIPY (**5**) (14 mg, 0.041 mmol), and $AgPF_6$ (11 mg, 0.041 mmol). Yield: 39 mg (78%). ^{1}H NMR (500 MHz, CD_2Cl_2) δ: 9.07 (d, *J* = 5.9 Hz, 1H, Ar*H*), 8.96 (d, *J* = 8.4 Hz, 1H, Ar*H*), 8.82 (d, *J* = 8.5 Hz, 1H, Ar*H*), 8.52 (s, 2H, Pyridine *H*), 8.26 (d, *J* = 8.1 Hz, 1H, Ar*H*), 8.18 (d, *J* = 7.7 Hz, 1H, Ar*H*), 8.07–7.98 (m, 3H, Ar*H*), 7.86 (dd, *J* = 17.9, 8.2 Hz, 3H, Ar*H*), 7.78 (t, *J* = 7.4 Hz, 1H, Ar*H*), 7.65 (d, *J* = 5.5 Hz, 1H, Ar*H*), 7.61 (d, *J* = 6.0 Hz, 1H, Ar*H*), 7.21 (s, 2H, Pyridine *H*), 7.17–7.11 (m, 2H, Ar*H*), 7.05 (dd, *J* = 17.1, 7.4 Hz, 3H, Ar*H*), 6.93 (d, *J* = 6.7 Hz, 1H, Ar*H*), 6.81 (t, *J* = 7.2 Hz, 1H, Ar*H*), 6.34 (d, *J* = 7.6 Hz, 1H, Ar*H*), 6.18 (d, *J* = 7.1 Hz, 1H, Ar*H*), 6.02 (d, *J* = 8.0 Hz, 2H, Pyrrole *H*), 4.37 (s, 2H, C*H*2), 2.44 (s, 6H, C*H*3), 2.06 (s, 3H, C*H*3), 2.01 (s, 9H, C*H*3). ^{19}F NMR (376 MHz, CD_2Cl_2) δ: −73.08 (d, *J* = 710.7 Hz, 6F, PF_6), −145.97 to −146.64 (m, 2F, BF_2). ^{11}B NMR (160 MHz, CD_2Cl_2) δ: −0.47 (t, *J* = 32.2 Hz, 1B, BF_2). HRMS (ESI): *m/z* calcd. for $C_{58}H_{49}BF_8IrN_6P$ $[M - PF_6]^+$: 1071.3709, found: 1071.4089.

X-ray Crystallography Procedures. Single crystals were grown by diffusion of hexane into concentrated CH_2Cl_2 solutions, in air. Crystals were mounted on a Bruker Apex II three-circle diffractometer using MoKα radiation (λ = 0.71073 Å). The data was collected at 123(2) K and was processed and refined within the APEXII software. Structures were solved by direct methods in SHELXS or by intrinsic phasing in SHELXT and refined by standard difference Fourier techniques in the program SHELXL.[6] Hydrogen atoms were placed in calculated positions using the standard riding model and refined isotropically; all non-hydrogen atoms were refined anisotropically. All three crystals (**6a**–**8a**) were partially desolvated, resulting in large regions of electron density corresponding to disordered and/or partially occupied dichloromethane solvent molecules. In the case of **6a**, the presence of large voids and the heavy disorder of the solvent necessitated the use of the SQUEEZE function in PLATON.[7] For **7a** and **8a**, the disordered solvent could be modeled, but it did result in high checkCIF alerts (Level B for **7a**, Level A and B for **8a**) for abnormally large chlorine ellipsoids. Notwithstanding these checkCIF errors, we concluded that the model which included the disordered solvent was satisfactory, and did not use SQUEEZE for these latter two structures. Distance restraints and rigid-bond restraints (SIMU and DELU) were used on all disordered parts. Crystallographic details of complexes **6a**–**8a** are summarized in Tables S1 in supporting information.

Table S1. Summary of X-ray crystallographic data for **6a**, **7a**, and **8a**.

	**6a**	**7a**•1.5CH_2Cl_2	**8a**•CH_2Cl_2
CCDC	1883526	1883527	1883528
Crystal data			
Chemical formula	$C_{49}H_{39}BF_{12}IrN_6P$	$C_{56.50}H_{46}BCl_3F_{12}IrN_6P$	$C_{51}H_{43}BCl_2F_{12}IrN_6P$
M_r	1173.84	1377.32	1272.79
Crystal system, space group	Monoclinic, $P2_1/c$	Triclinic, $P\bar{1}$	Monoclinic, $C2/c$
Temperature (K)	123	123	123
a, b, c (Å)	18.512(5), 18.686(5), 32.097(8)	10.868(3), 14.021(3), 20.638(5)	45.386(6), 11.4399(14), 20.573(3)
α, β, γ (°)	90, 91.307(3), 90	105.005(3), 92.613(3), 95.539(3)	90, 102.781(1), 90
V (Å^3)	11100(5)	3015.1(13)	10417(2)
Z	8	2	8
μ (mm^{-1})	2.51	2.45	2.78
Crystal size (mm)	0.29 × 0.17 × 0.04	0.19 × 0.16 × 0.08	0.35 × 0.27 × 0.12
Data collection			
T_{min}, T_{max}	0.516, 0.746	0.562, 0.746	0.561, 0.746
No. of measured, independent and observed [$I > 2\sigma(I)$] reflections	56882, 23916, 17632	16782, 12744, 10915	54925, 11935, 10920
R_{int}	0.055	0.020	0.025
$(\sin \theta/\lambda)_{max}$ (Å^{-1})	0.641	0.641	0.650
Refinement			
$R[F^2 > 2\sigma(F^2)]$, $wR(F^2)$, S	0.076, 0.242, 1.11	0.084, 0.311, 1.39	0.062, 0.154, 1.23
No. of reflections	23916	12744	11935
No. of parameters	1328	853	730
No. of restraints	2337	1261	756
	$w = 1/[\sigma^2(F_o^2) + (0.1031P)^2 + 138.2186P]$ where $P = (F_o^2 + 2F_c^2)/3$		$w = 1/[\sigma^2(F_o^2) + (0.0507P)^2 + 171.0678P]$ where $P = (F_o^2 + 2F_c^2)/3$
$\Delta\rho_{max}$, $\Delta\rho_{min}$ (e Å^{-3})	3.30, −3.20	5.85, −3.61	4.52, −3.43

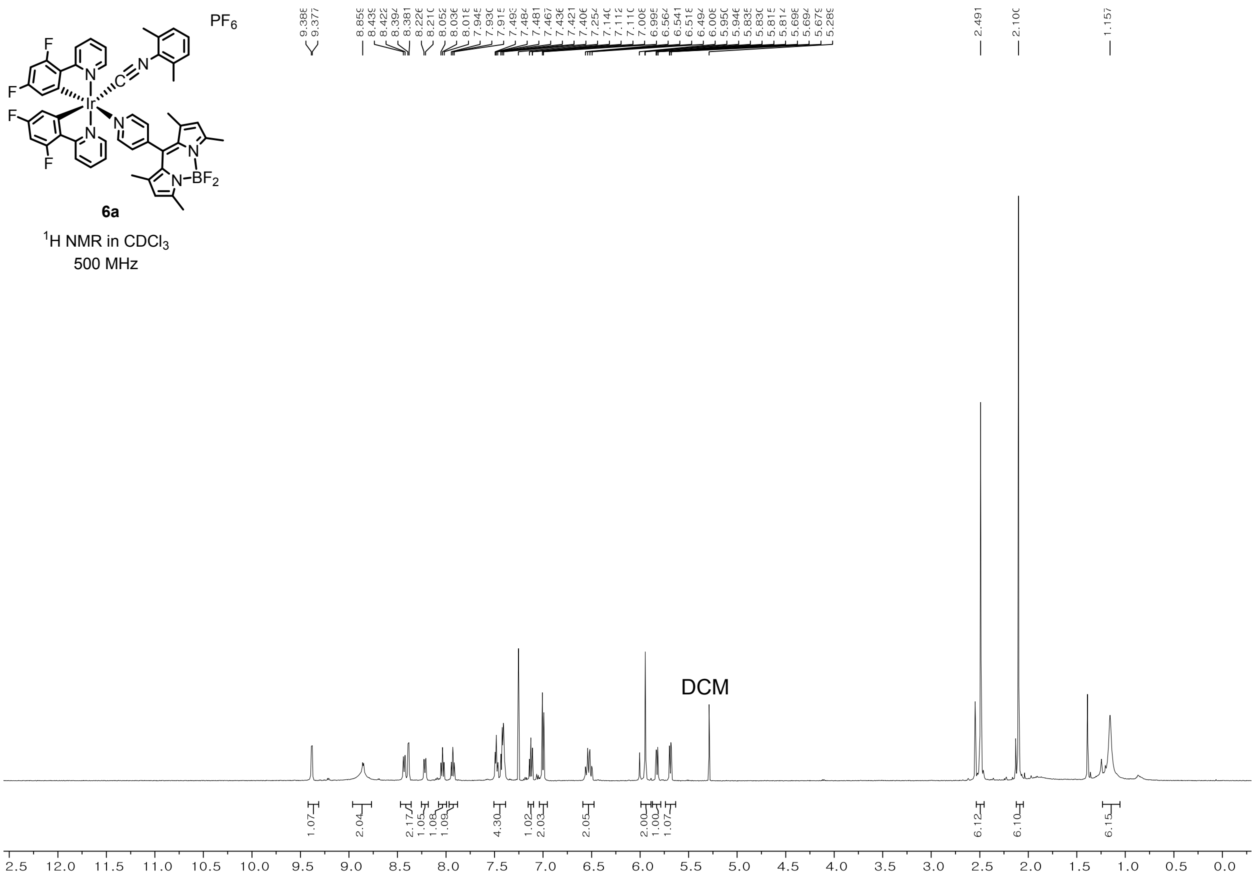

Fig. S1. ^{1}H NMR spectrum of complex **6a**, recorded at 500 MHZ in $CDCl_3$.

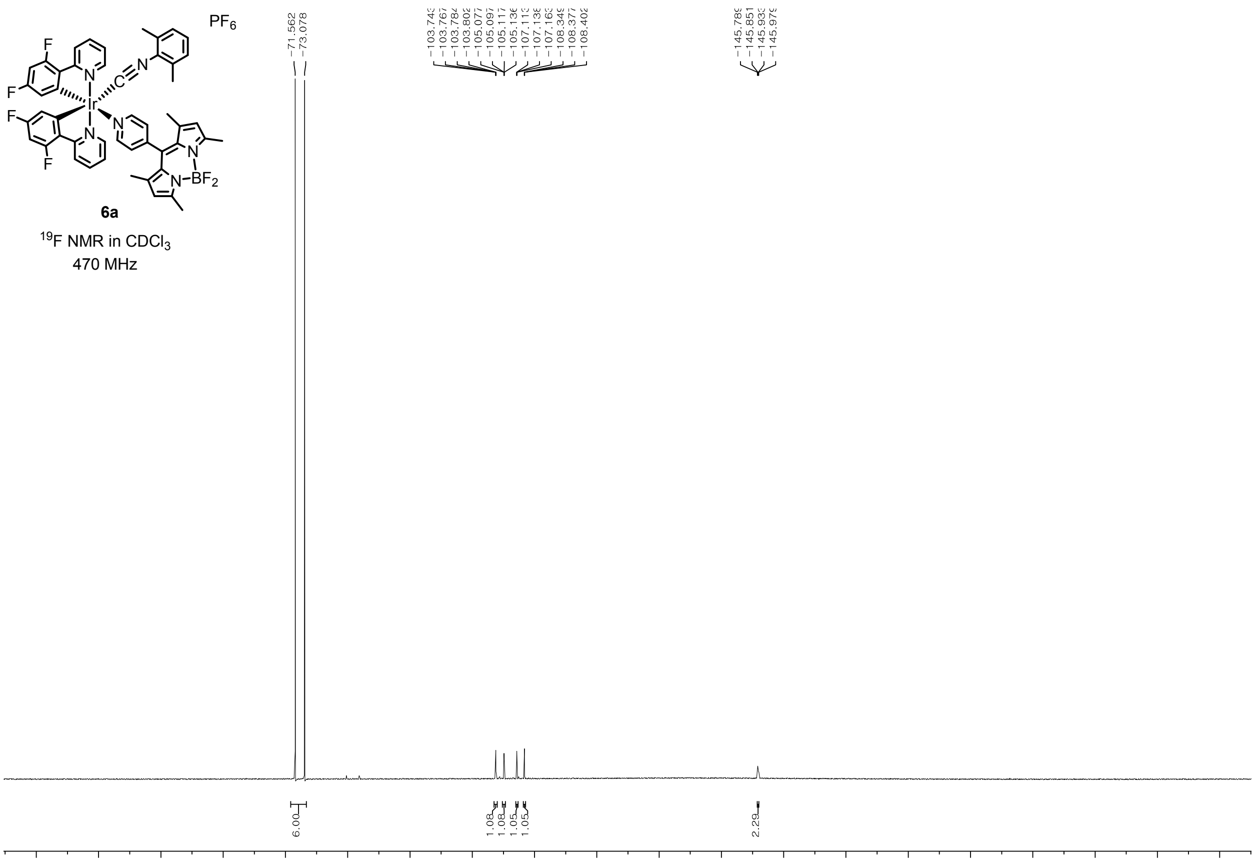

Fig. S2. ^{19}F NMR spectrum of complex **6a**, recorded at 470 MHZ in $CDCl_3$.

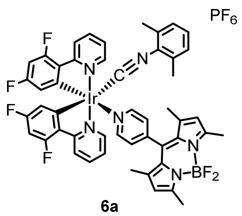

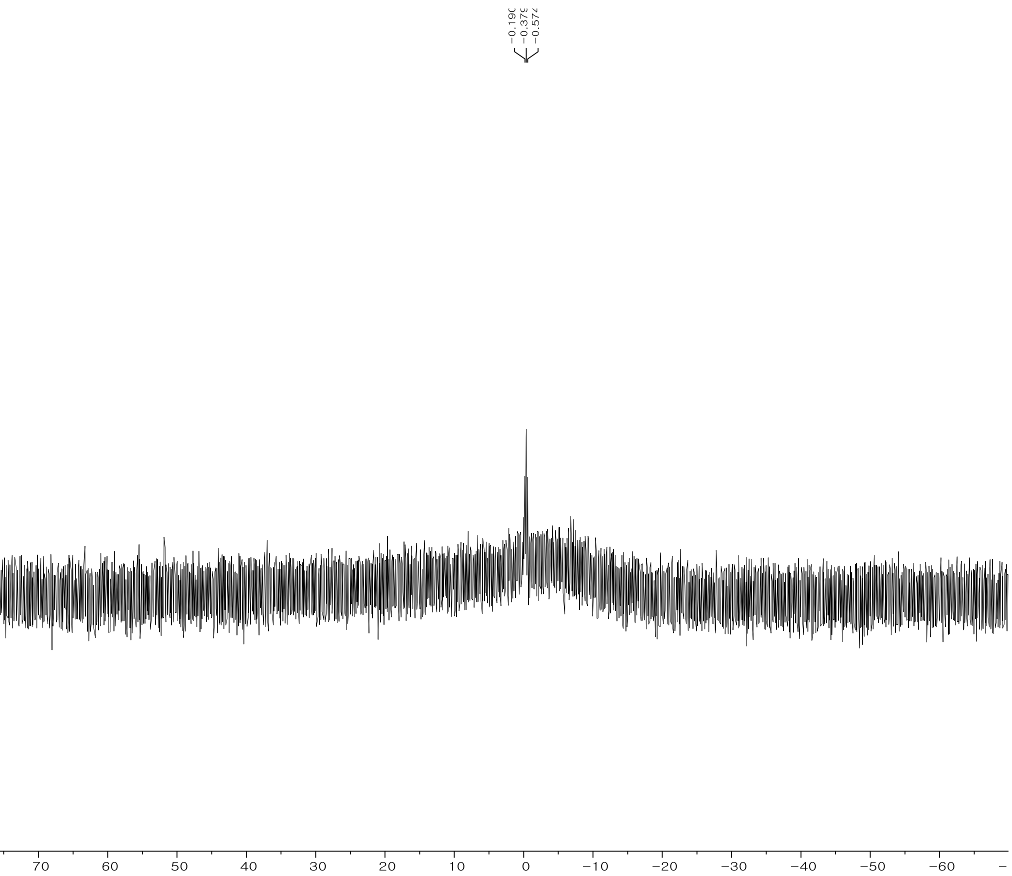

Fig. S3. ^{11}B NMR spectrum of complex **6a**, recorded at 160 MHZ in $CDCl_3$.

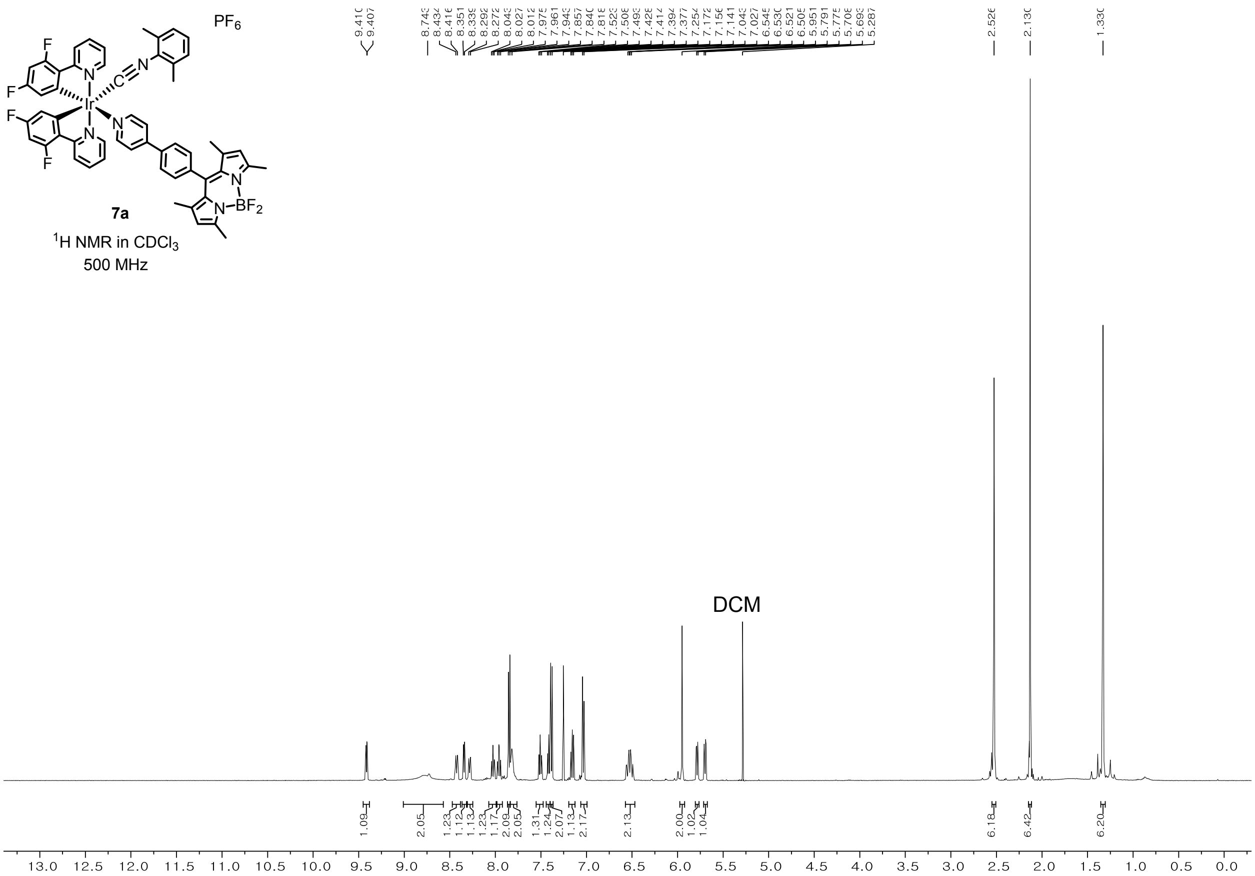

Fig. S4. ^{1}H NMR spectrum of complex **7a**, recorded at 500 MHZ in $CDCl_3$.

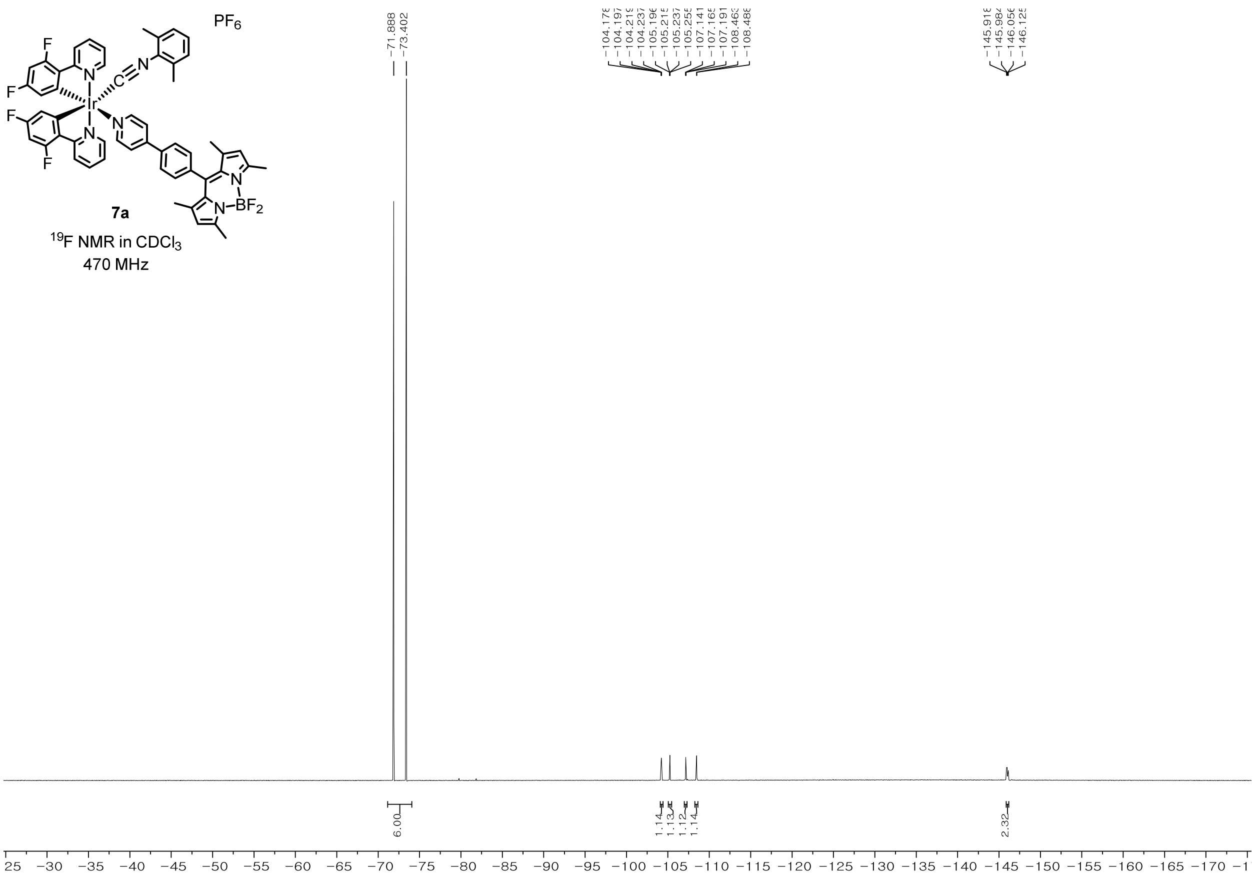

Fig. S5. ^{19}F NMR spectrum of complex **7a**, recorded at 470 MHZ in $CDCl_3$.

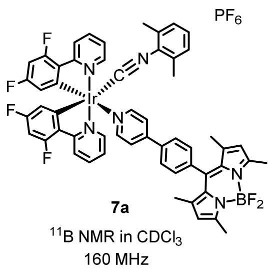

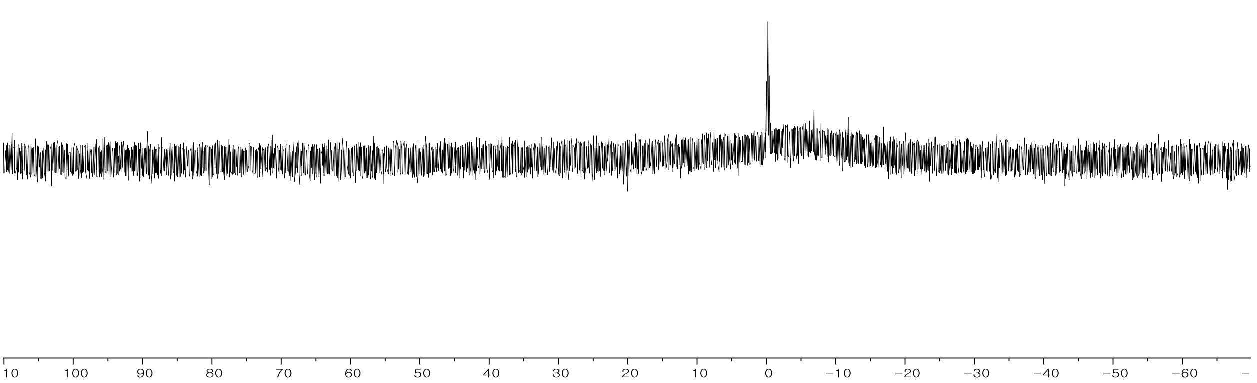

Fig. S6. ^{11}B NMR spectrum of complex **7a**, recorded at 160 MHZ in $CDCl_3$.

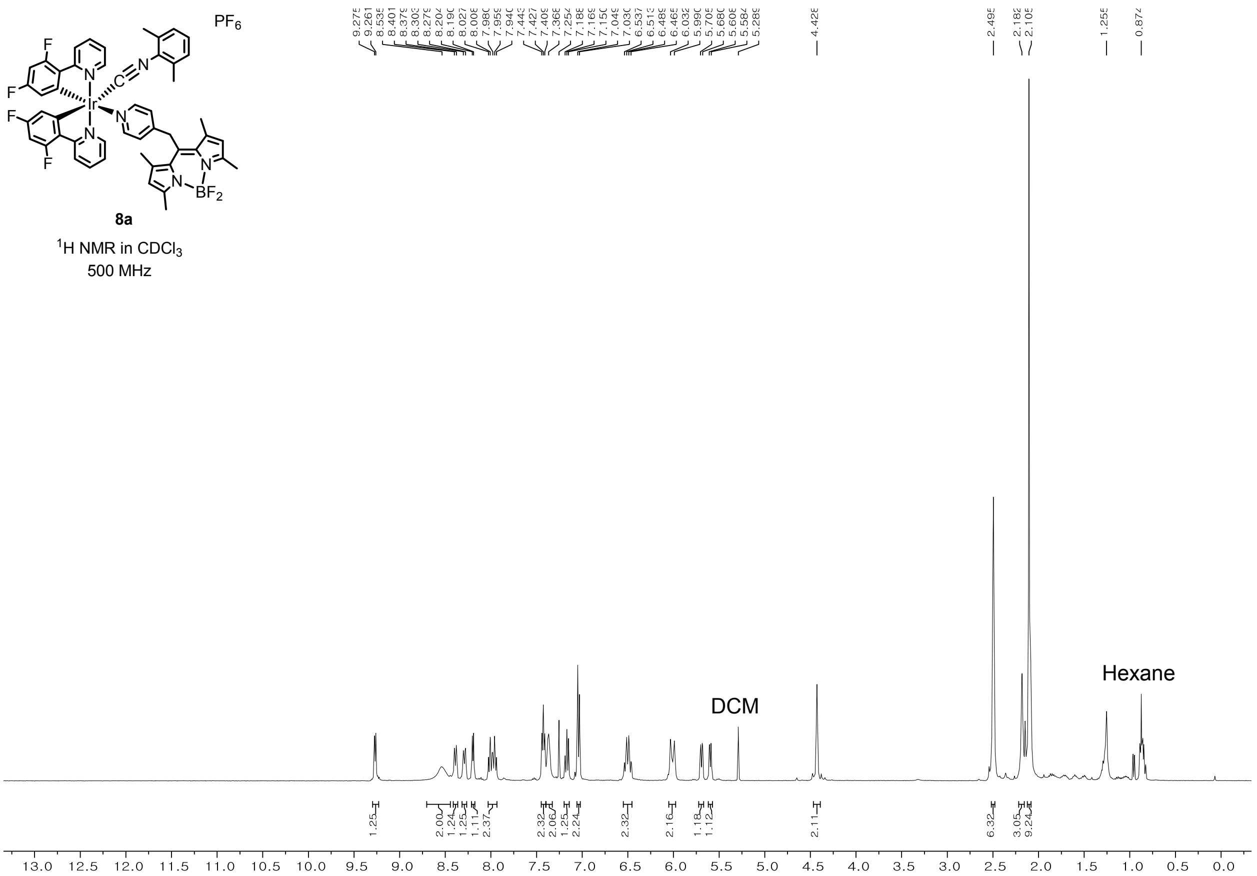

Fig. S7. ^{1}H NMR spectrum of complex **8a**, recorded at 400 MHZ in $CDCl_3$.

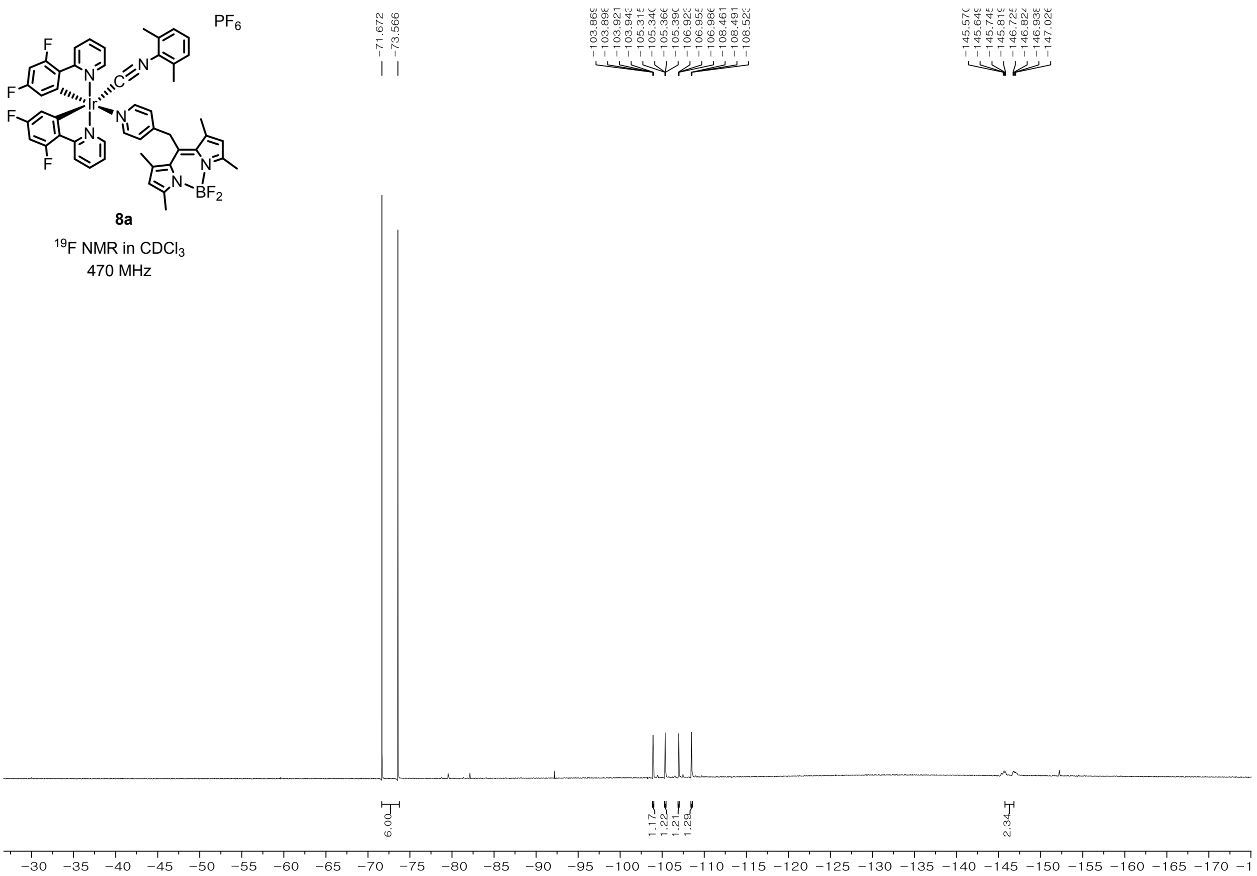

Fig. S8. ^{19}F NMR spectrum of complex **8a**, recorded at 376 MHZ in CDCl3.

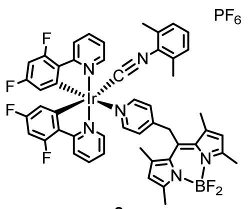

8a

^{11}B NMR in $CDCl_3$
160 MHz

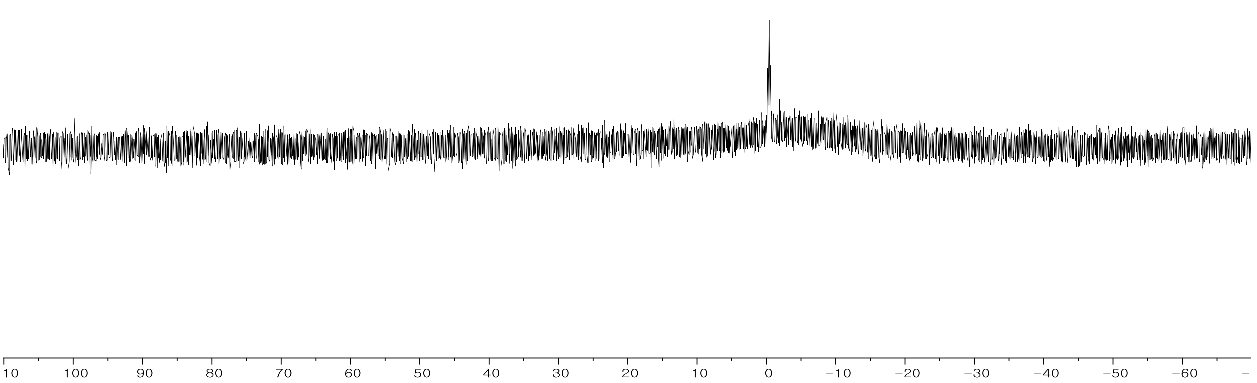

Fig. S9. ^{11}B NMR spectrum of complex **8a**, recorded at 160 MHZ in $CDCl_3$.

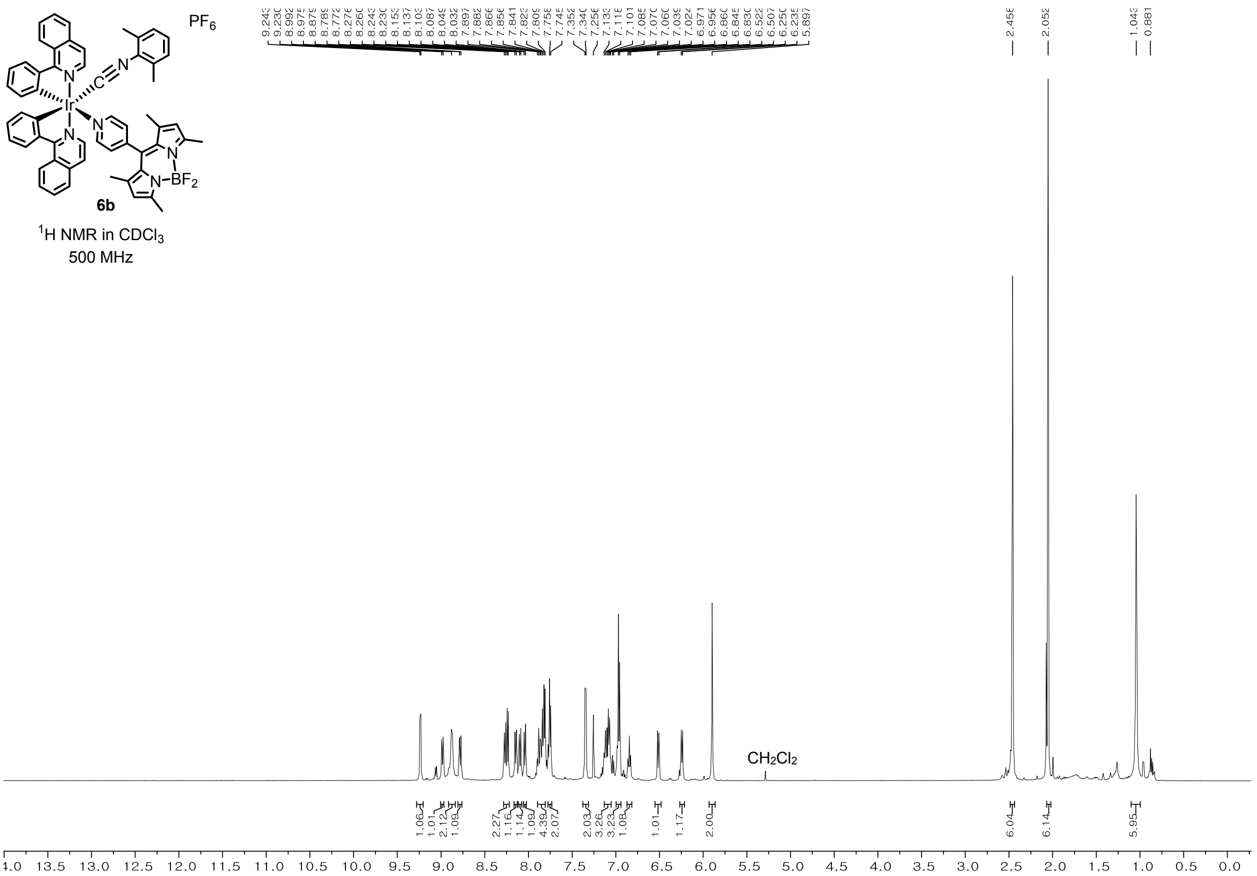

Fig. S10. ^{1}H NMR spectrum of complex **6b**, recorded at 500 MHZ in $CDCl_3$.

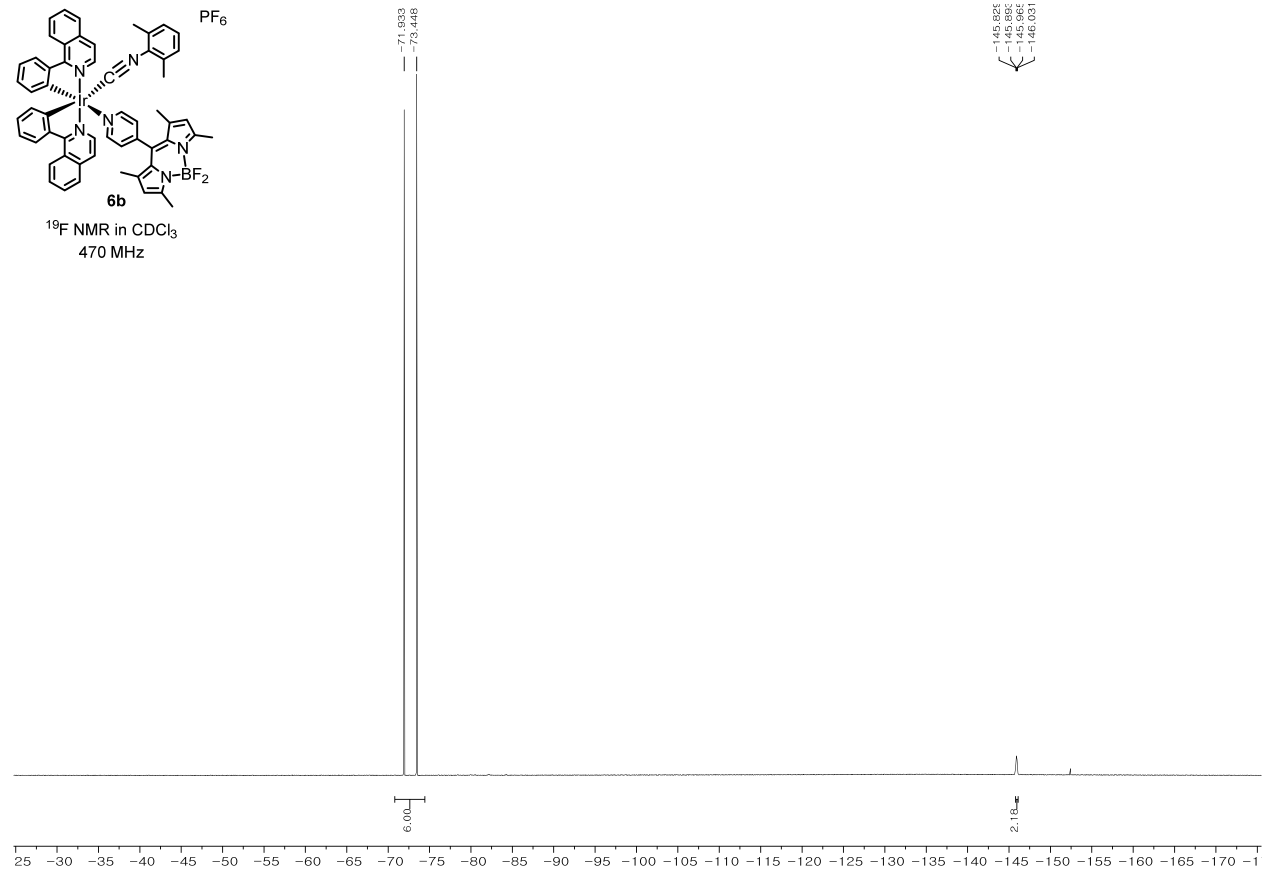

Fig. S11. ^{19}F NMR spectrum of complex **6b**, recorded at 470 MHZ in $CDCl_3$.

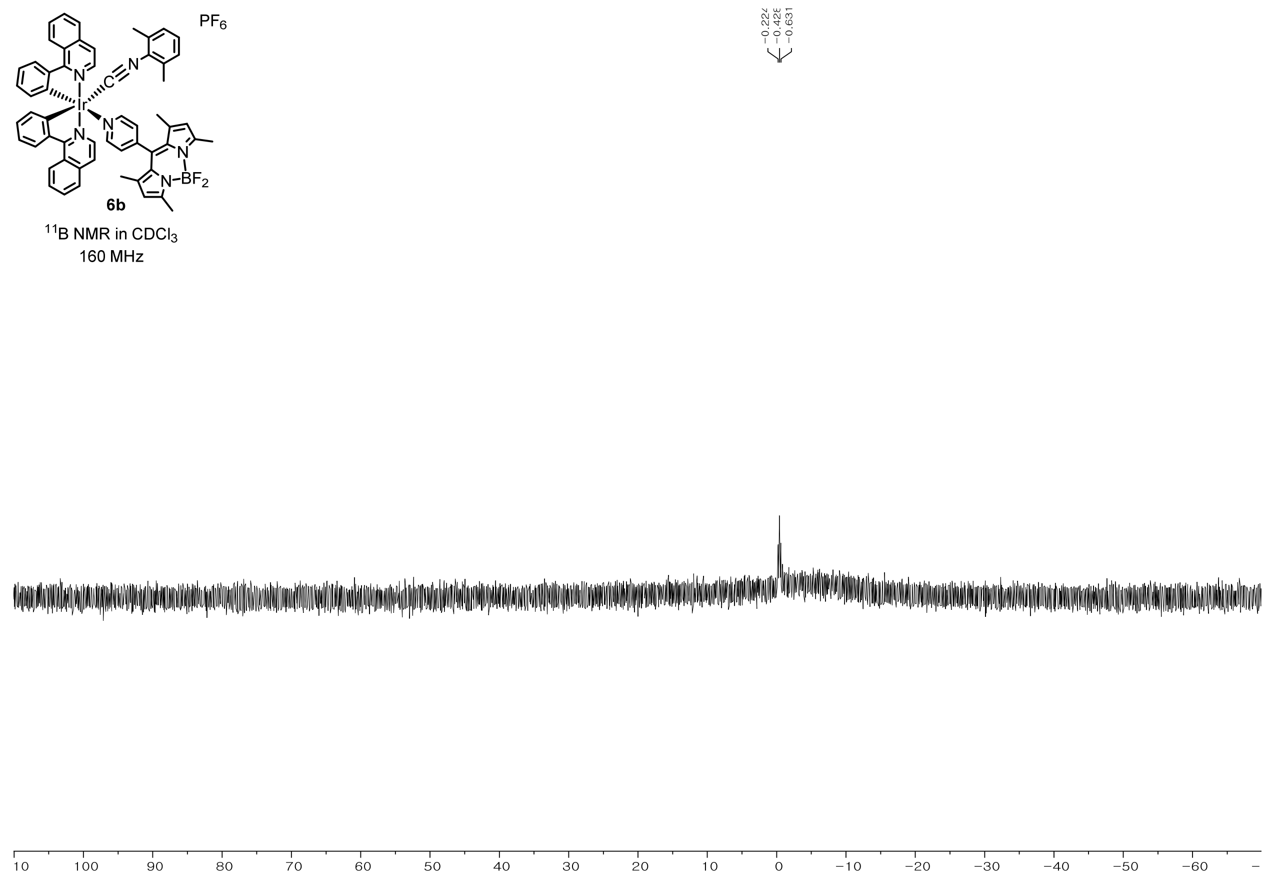

Fig. S12. ^{11}B NMR spectrum of complex **6b**, recorded at 160 MHZ in $CDCl_3$.

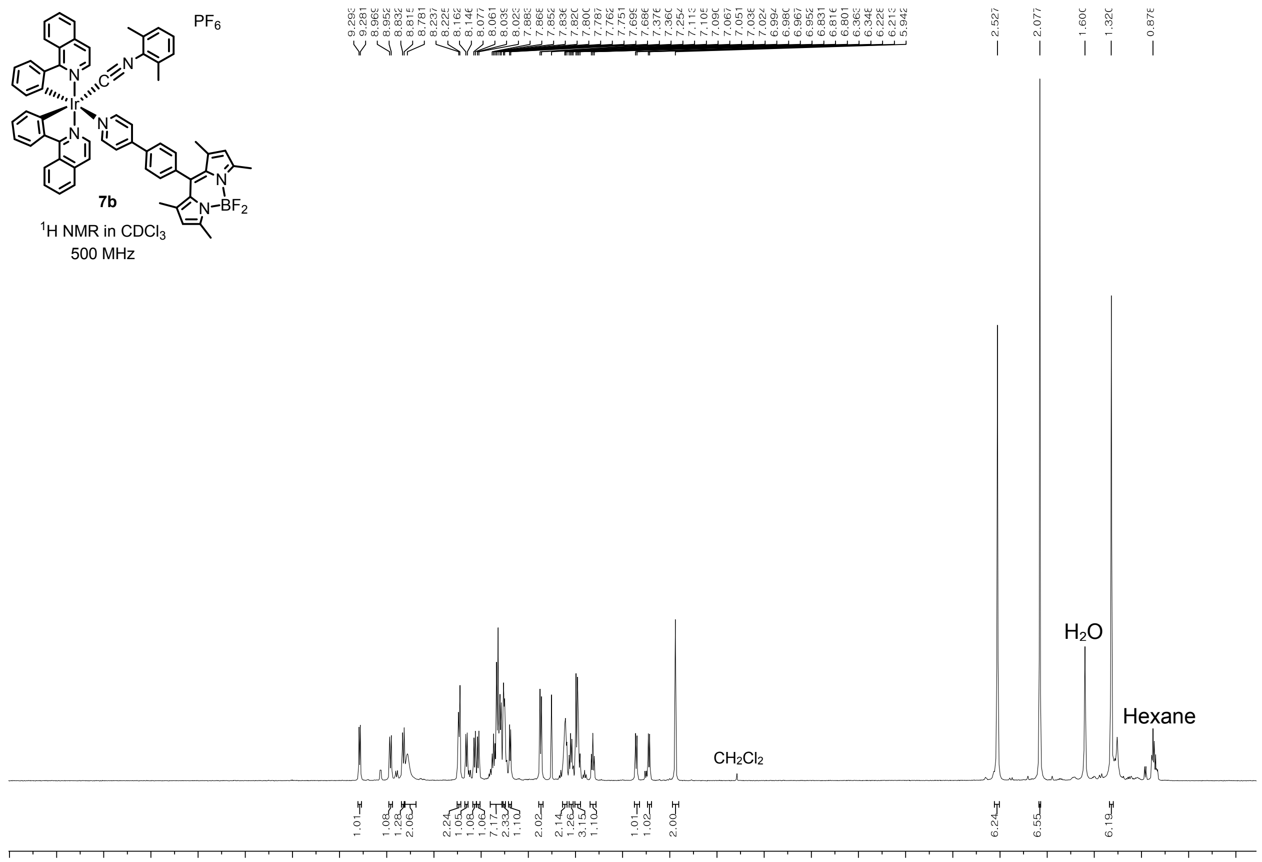

Fig. S13. ^{1}H NMR spectrum of complex **7b**, recorded at 500 MHZ in $CDCl_3$.

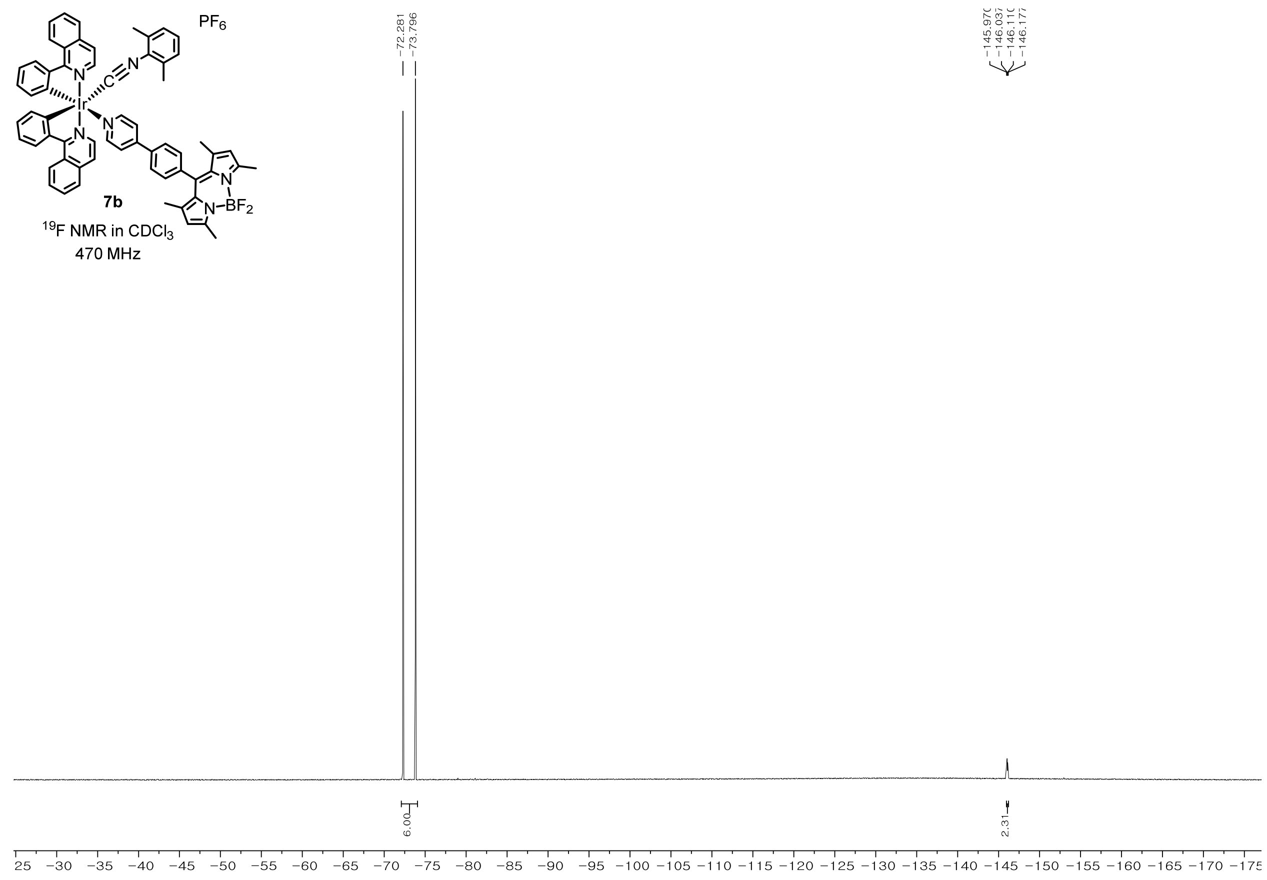

Fig. S14. ^{19}F NMR spectrum of complex **7b**, recorded at 470 MHZ in CDCl3.

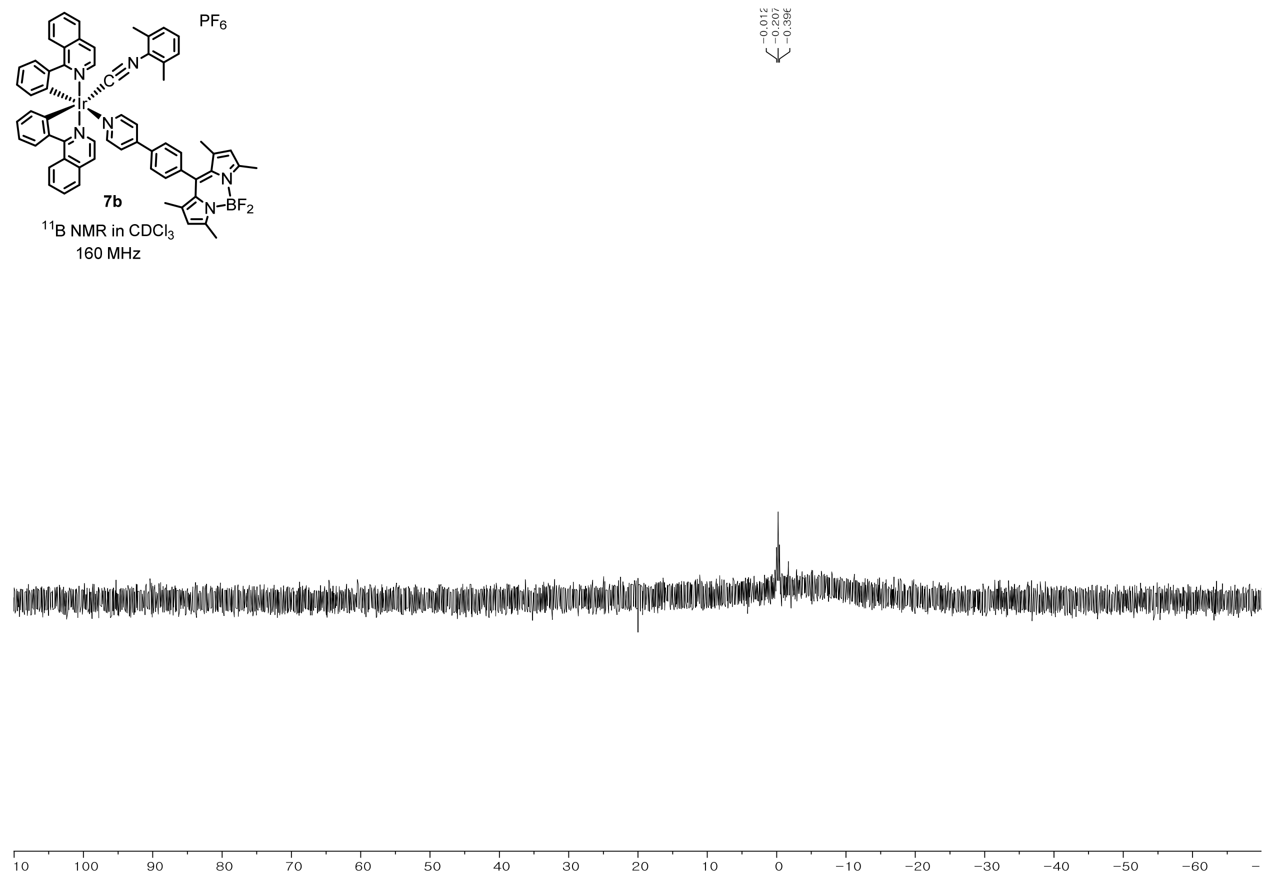

Fig. S15. ^{11}B NMR spectrum of complex **7b**, recorded at 160 MHZ in CDCl3.

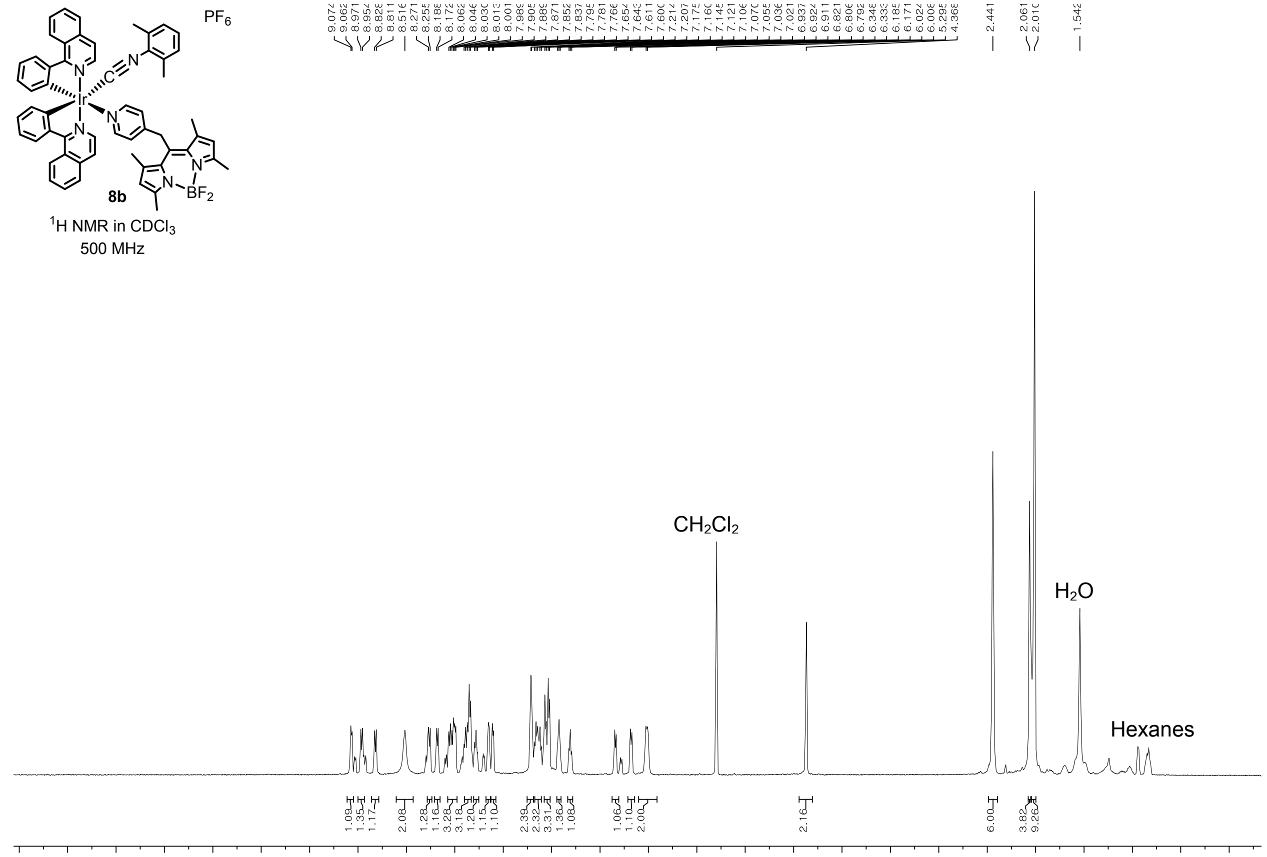

Fig. S16. ^{1}H NMR spectrum of complex **8b**, recorded at 500 MHZ in CD_2Cl_2.

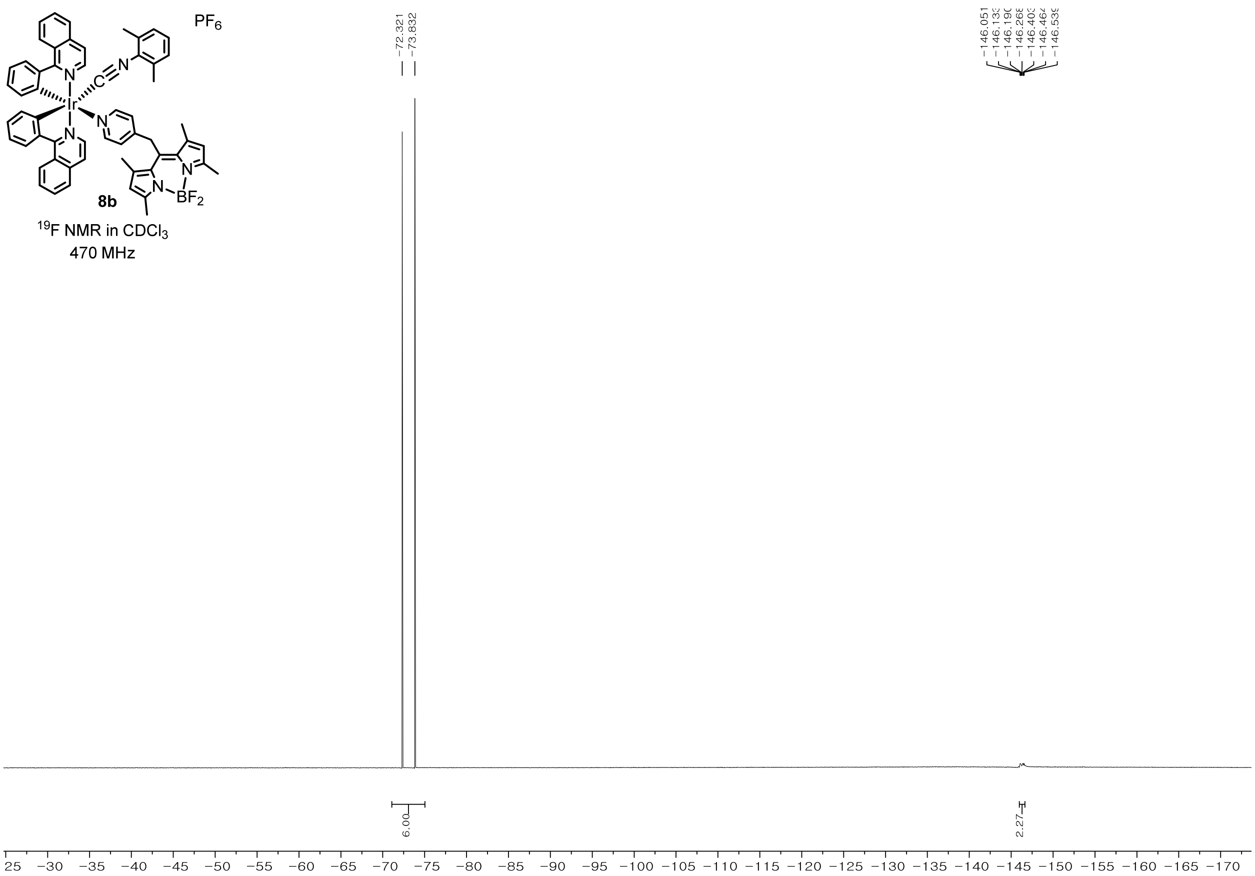

Fig. S17. ^{19}F NMR spectrum of complex **8b**, recorded at 470 MHZ in $CDCl_3$.

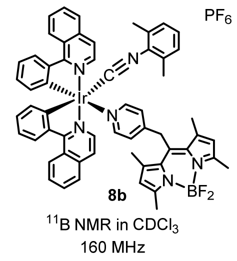

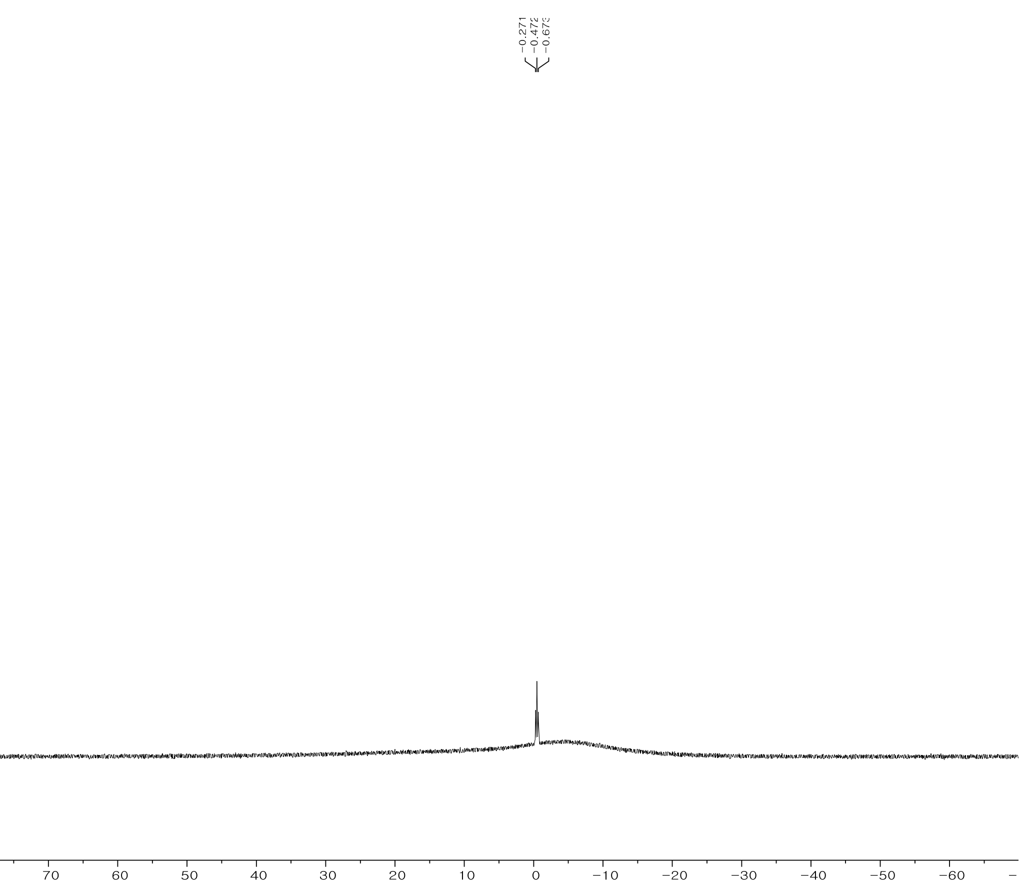

Fig. S18. ^{11}B NMR spectrum of complex **8b**, recorded at 160 MHZ in $CDCl_3$.

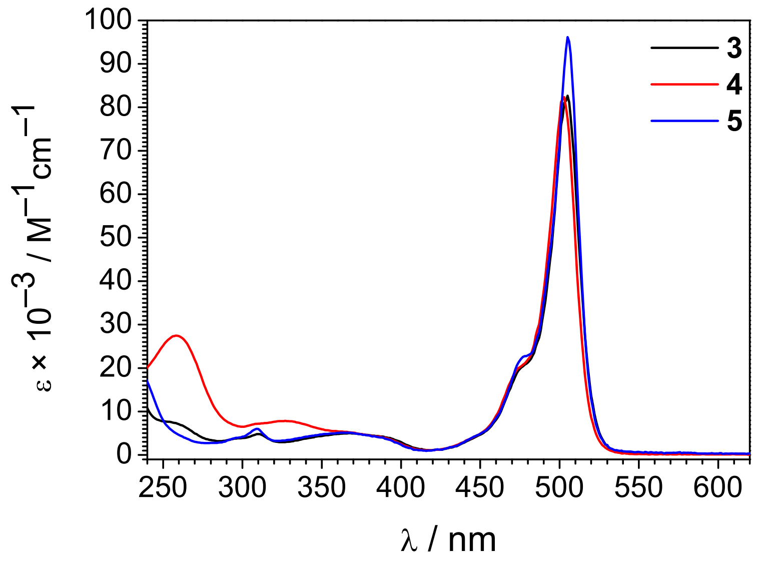

Fig. S19. Overlaid UV-vis absorption spectra of BODIPYs **3**–**5**. Absorption spectra were recorded at room temperature in CH_2Cl_2.

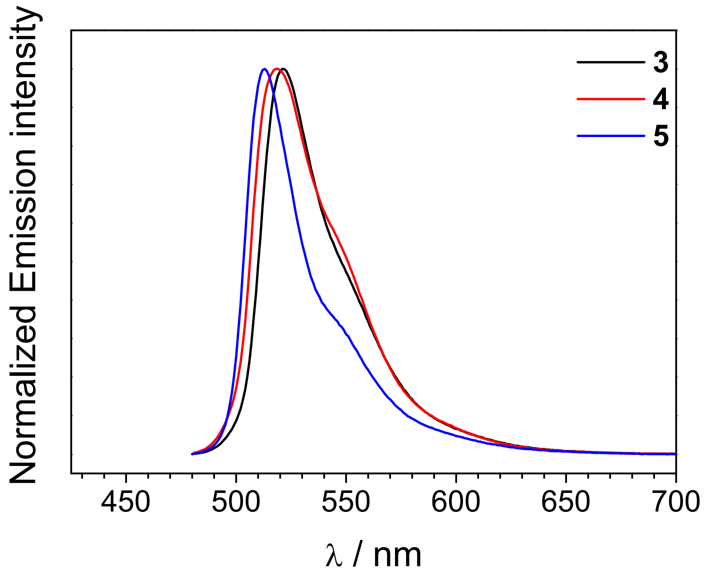

Fig. S20. Overlaid emission spectra of BODIPYs **3**–**5** were recorded at room temperature in CH_2Cl_2. Excitation for the emission spectra was at 475 nm.

Table S2. Summary of absorption and emission data for BODIPYs **3**–**5**.

	λ_{abs}/nm (ε × 10^{-3} /M^{-1} cm^{-1})	λ_{em}/nm	Φ	τ/ns	$k_r \times 10^{-8}/s^{-1}$	$k_{nr} \times 10^{-8}/s^{-1}$
**3**	505 (82)	521	0.30	1.9	160	370
**4**	503 (82)	518	0.43	2.7	160	210
**5**	506 (96)	514	0.99	6.6	150	1.5

The UV-vis absorption and emission spectra were measured in CH_2Cl_2 at 293 K, and the samples were excited at 475 nm for steady-state measurements and 455 nm for lifetimes.

Table S3. Summary of UV-vis absorption data for Ir-BODIPY constructs **6**–**8**.

	λ_{abs}/nm (ε × 10^{-3} /M^{-1} cm^{-1})
**6a**	255 (51), 311 (sh) (24), 351 (sh) (10), 510 (65)
**7a**	260 (58), 314 (sh) (36), 504 (70)
**8a**	256 (56), 309 (26), 349 (sh) (9.4), 508 (66)
**6b**	280 (31), 352 (12), 400 (sh) (6.1), 509 (17)
**7b**	281 (29), 350 (sh) (11), 401 (sh) (5.1), 504 (12)
**8b**	280 (30), 351 (11), 399 (sh) (5.9), 508 (13)

UV-vis absorption spectra were measured in CH_2Cl_2 at 293 K.

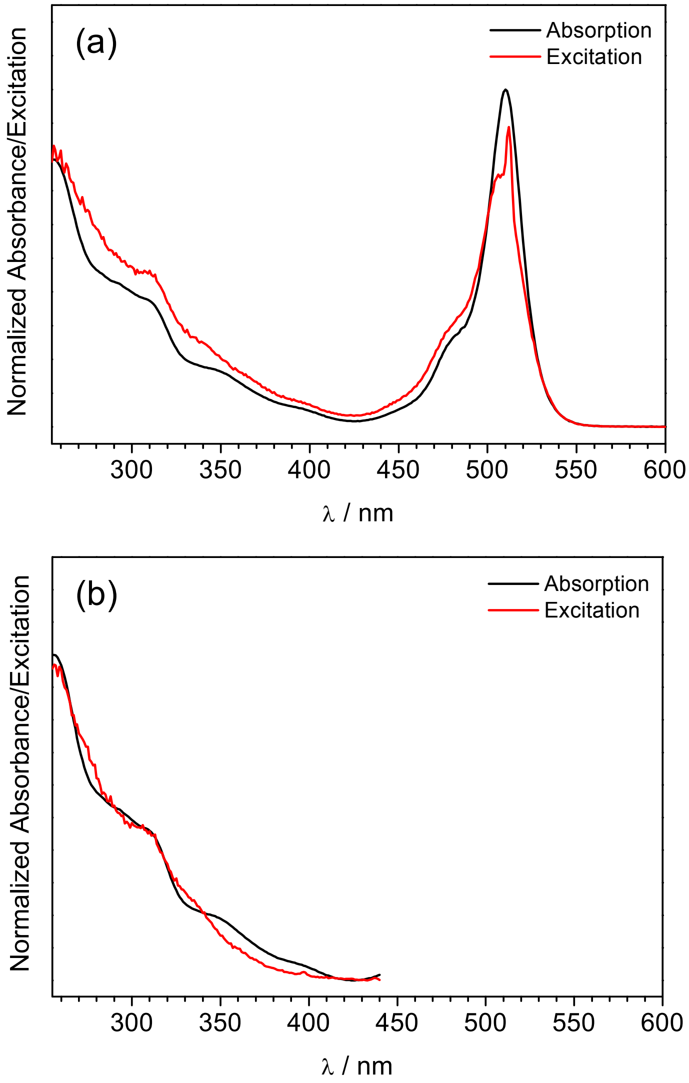

Fig. S21. Overlaid UV-vis absorption and excitation spectra of complex **6a**, recorded in CH_2Cl_2 at room temperature. For the excitation spectrum, (a) λ_{em} = 510 nm, (b) λ_{em} = 450 nm.

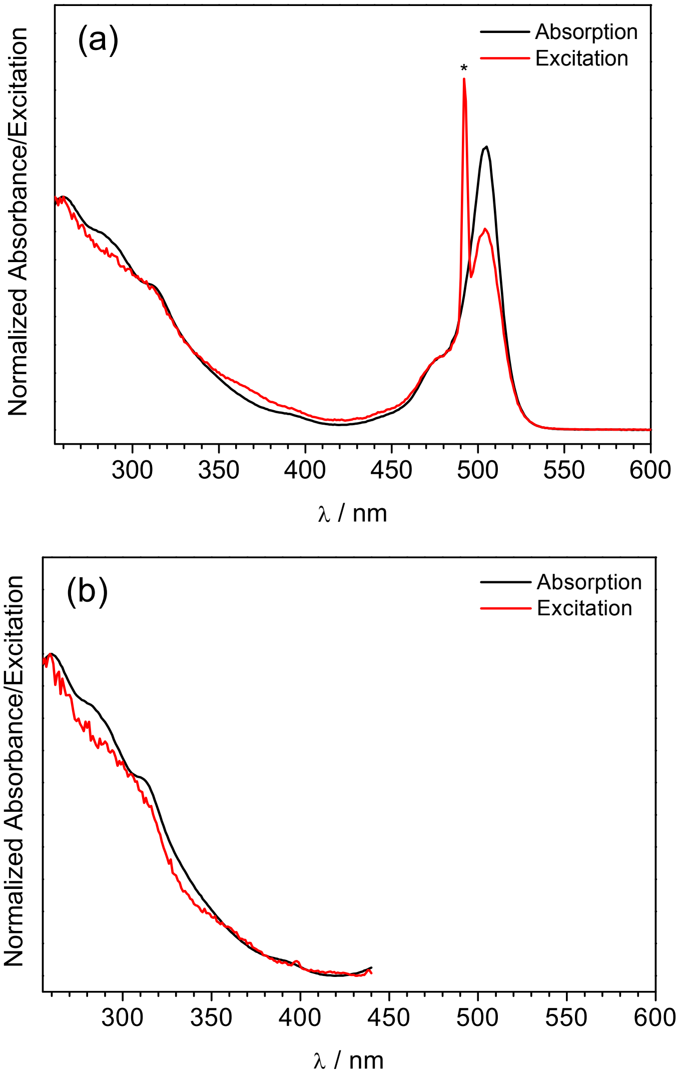

Fig. S22. Overlaid UV-vis absorption and excitation spectra of complex **7a**, recorded in CH_2Cl_2 at room temperature. For the excitation spectrum, (a) $\lambda_{em} = 490$ nm, (b) $\lambda_{em} = 450$ nm,. The * marks a feature caused by scattered excitation light at the detection wavelength

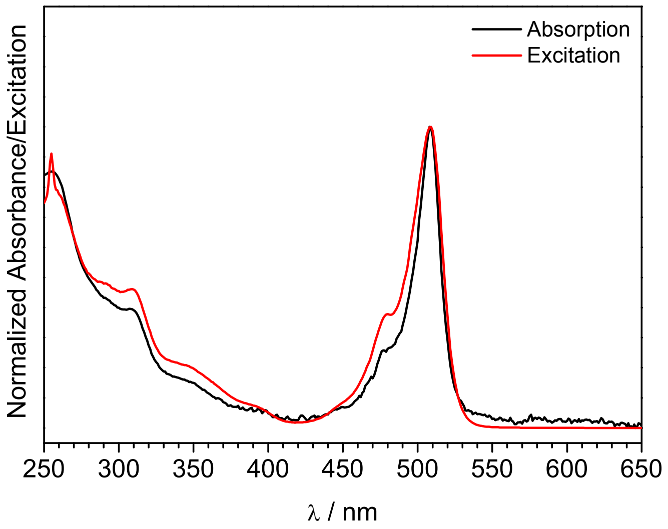

Fig. S23. Overlaid UV-vis absorption and excitation spectra of complex **8a**, recorded in CH_2Cl_2 at room temperature. For the excitation spectrum, λ_{em} = 510 nm.

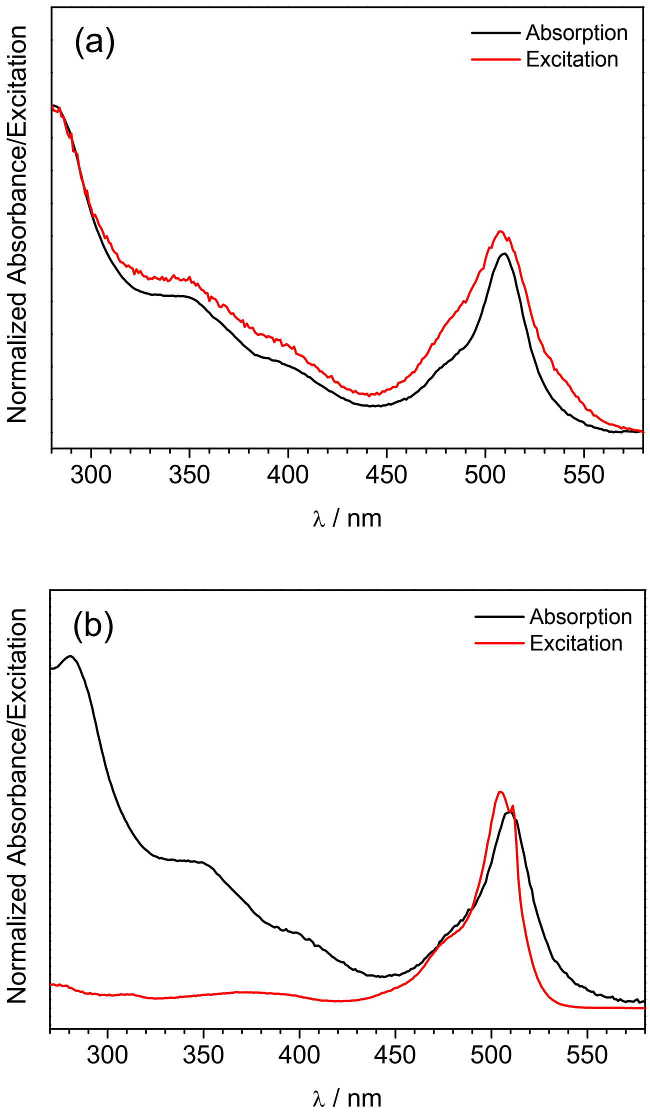

Fig. S24. Overlaid UV-vis absorption and excitation spectra of complex **6b**, recorded in CH_2Cl_2 at room temperature. For the excitation spectrum, (a) λ_{em} = 600 nm, (b) λ_{em} = 510 nm.

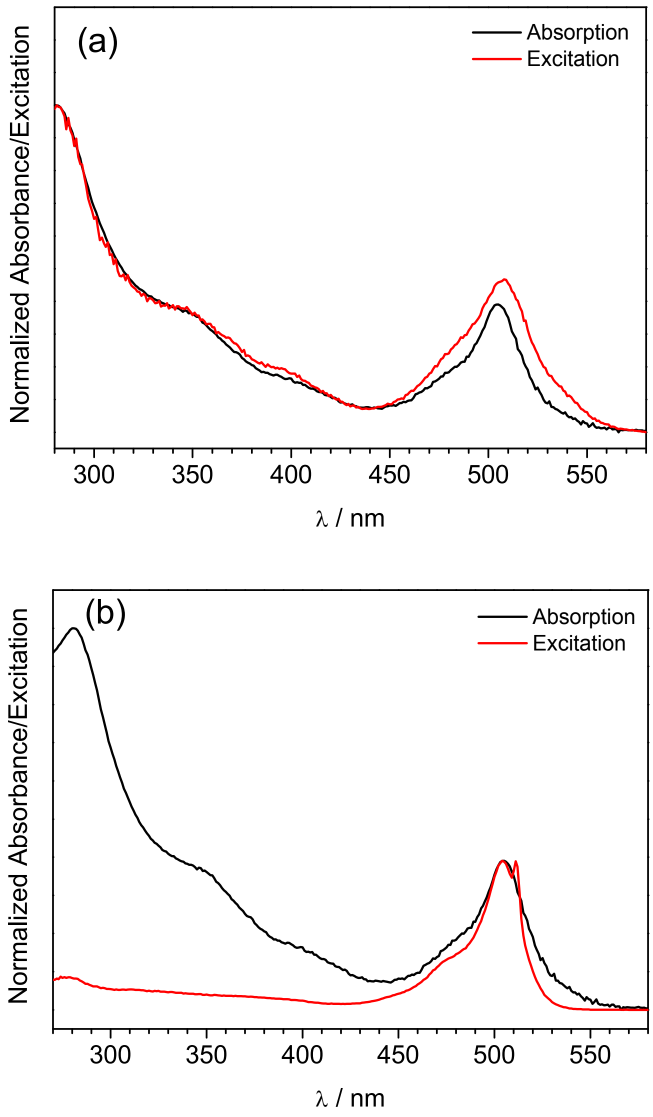

Fig. S25. Overlaid UV-vis absorption and excitation spectra of complex **7b**, recorded in CH_2Cl_2 at room temperature. For the excitation spectrum, (a) λ_{em} = 600 nm, (b) λ_{em} = 510 nm.

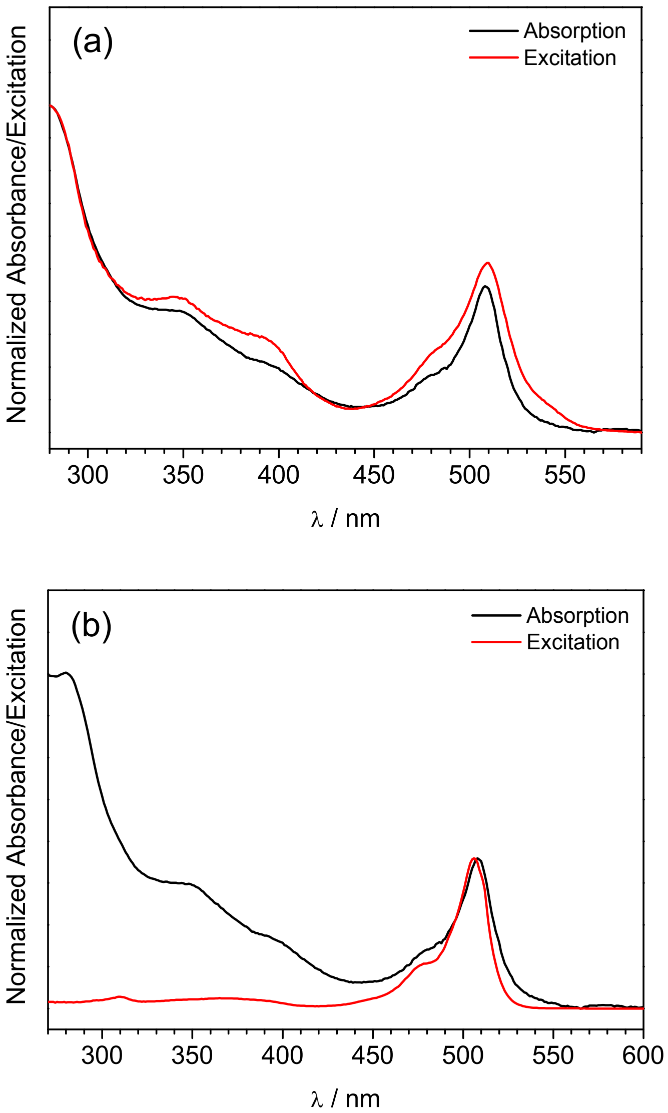

Fig. S26. Overlaid UV-vis absorption and excitation spectra of complex **8b**, recorded in CH_2Cl_2 at room temperature. For the excitation spectrum, (a) λ_{em} = 600 nm, (b) λ_{em} = 510 nm.

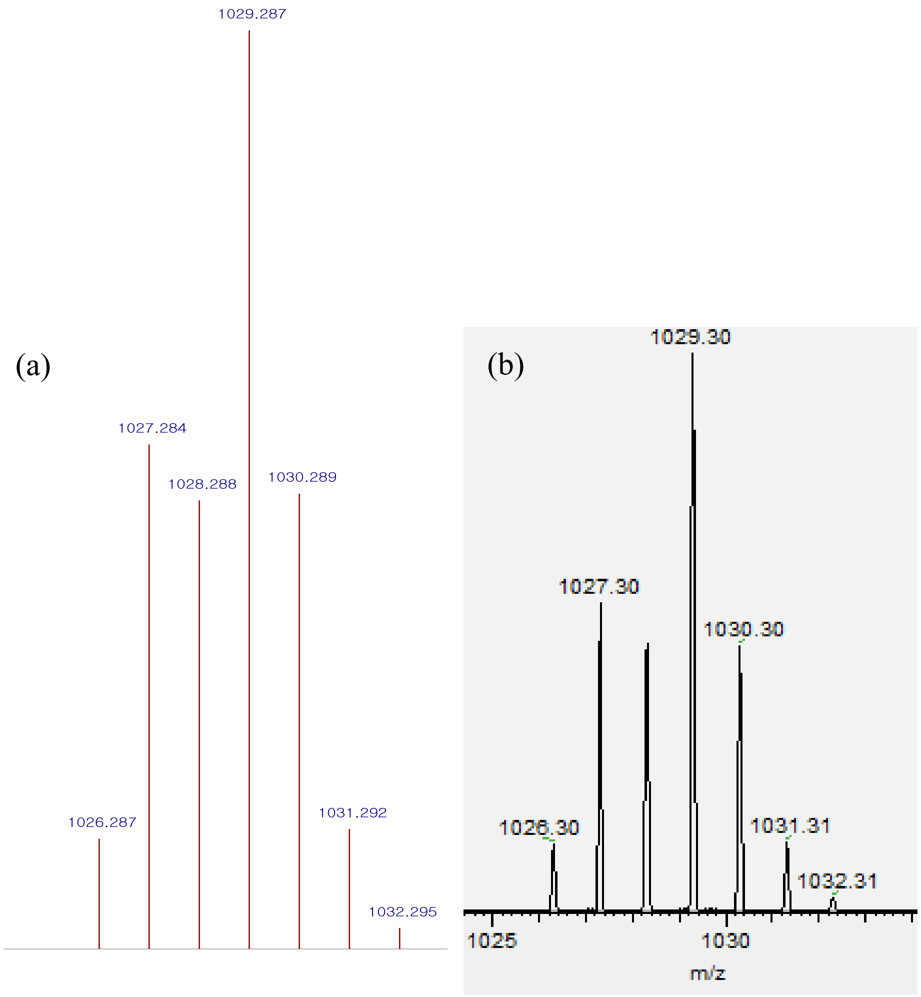

Fig. S27. Simulated (a) and experimental (b) isotropic distribution patterns of complex **6a**: the molecular ion peak ($[M - PF_6]^+$).

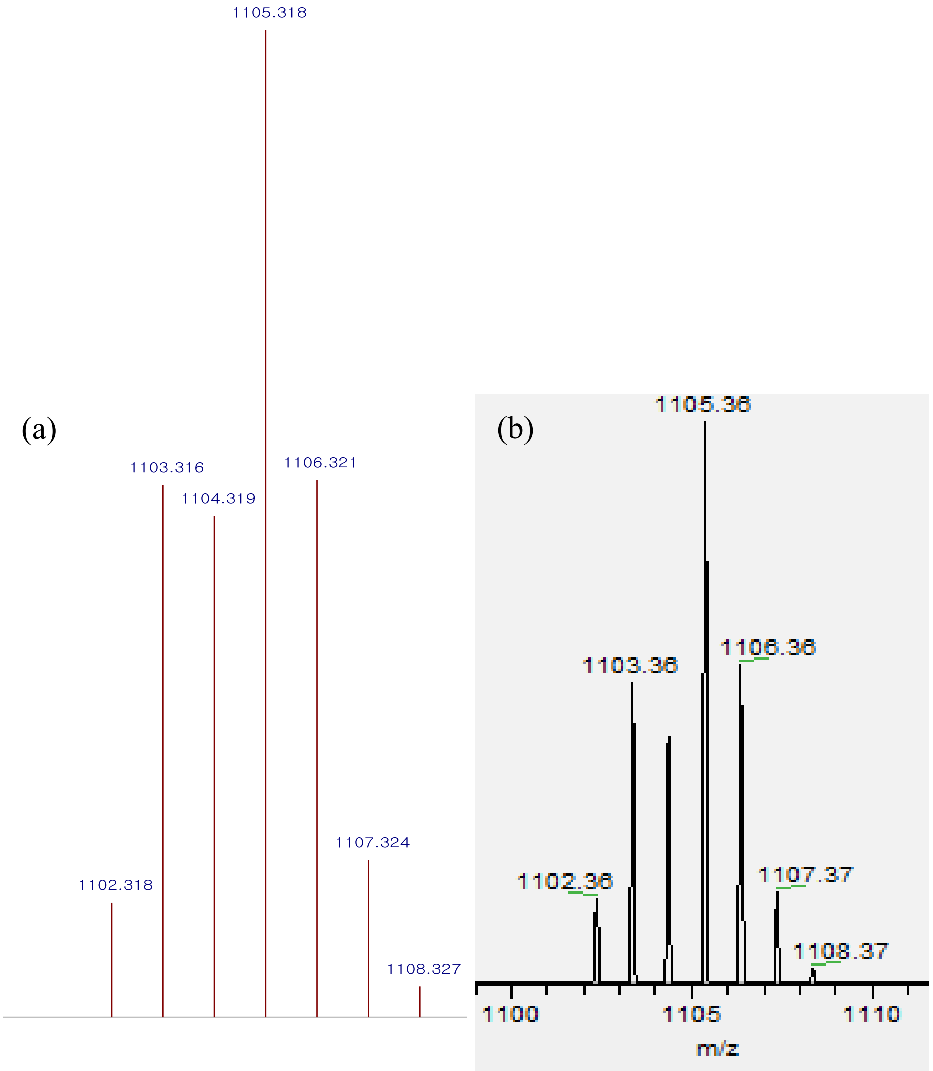

Fig. S28. Simulated (a) and experimental (b) isotropic distribution patterns of complex **7a**: the molecular ion peak ($[M - PF_6]^+$).

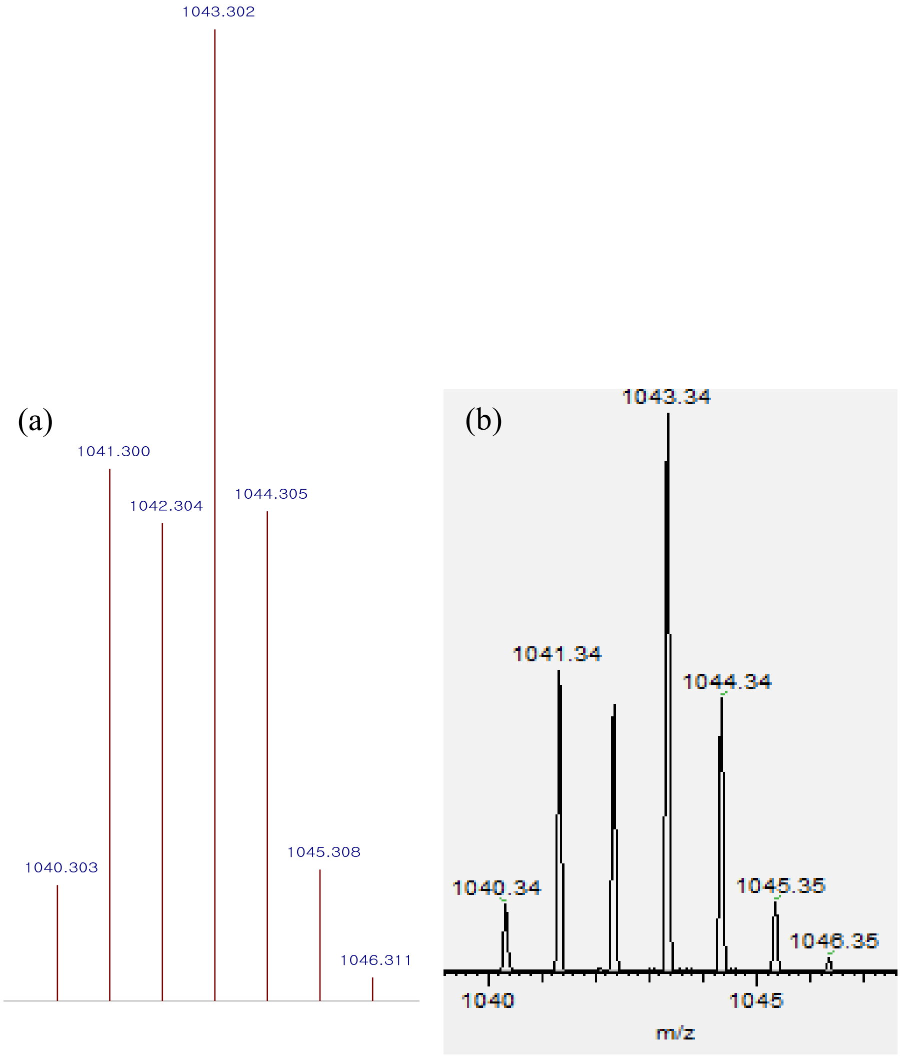

Fig. S29. Simulated (a) and experimental (b) isotropic distribution patterns of complex **8a**: the molecular ion peak ($[M - PF_6]^+$).

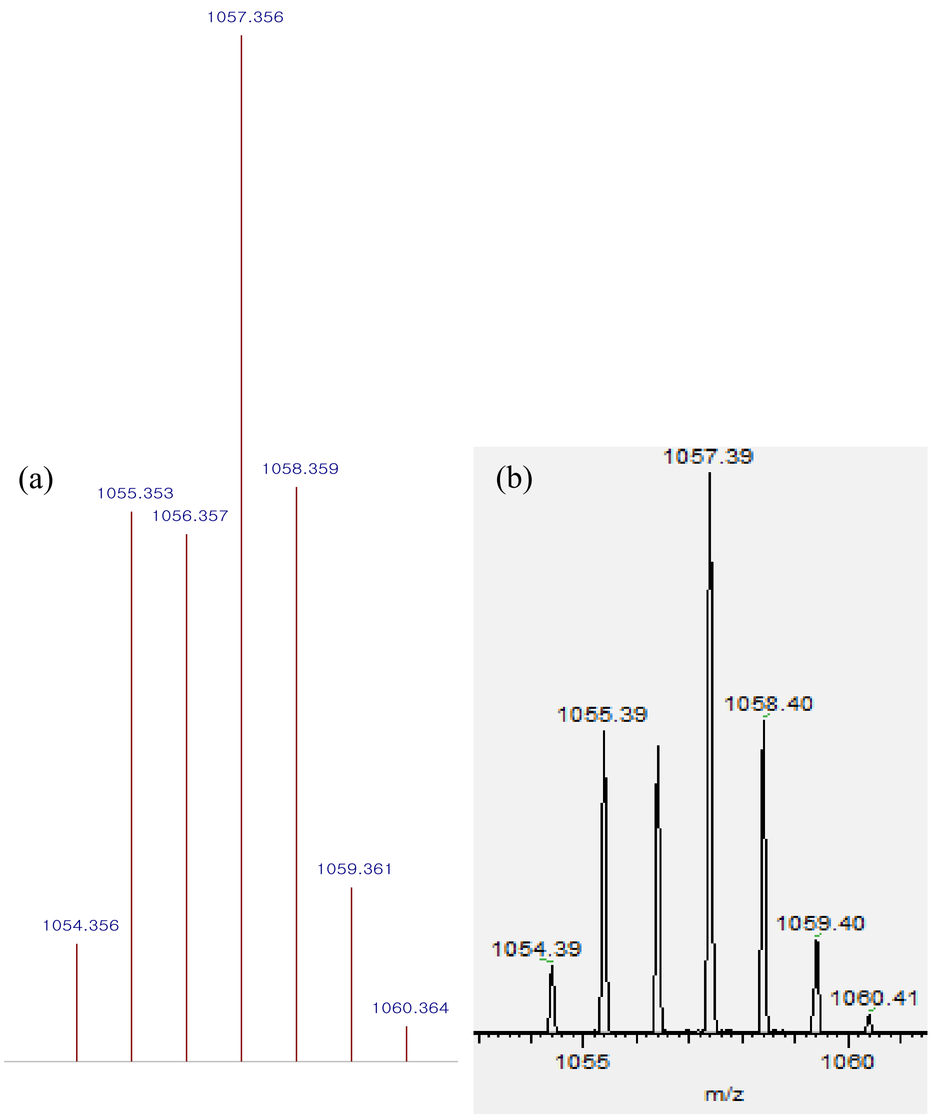

Fig. S30. Simulated (a) and experimental (b) isotropic distribution patterns of complex **6b**: the molecular ion peak ($[M - PF_6]^+$).

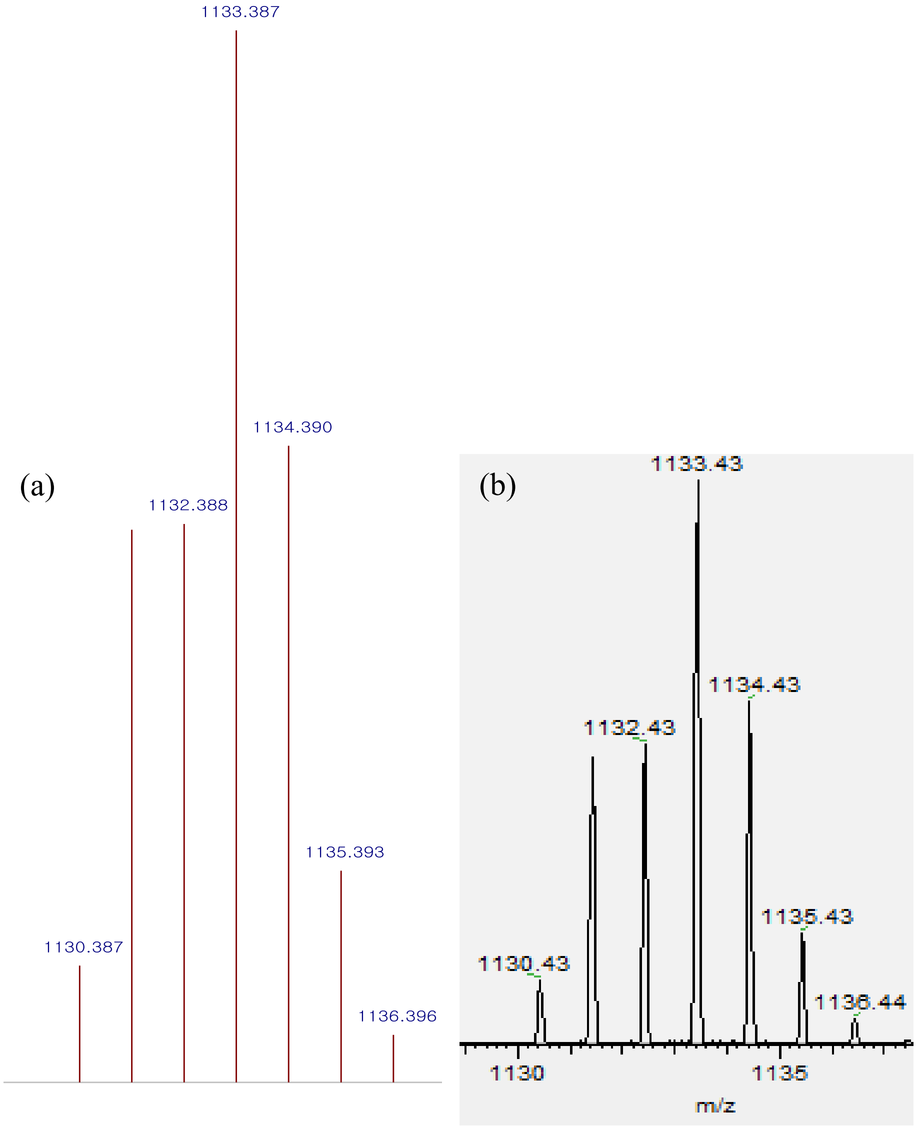

Fig. S31. Simulated (a) and experimental (b) isotropic distribution patterns of complex **7b**: the molecular ion peak ($[M - PF_6]^+$).

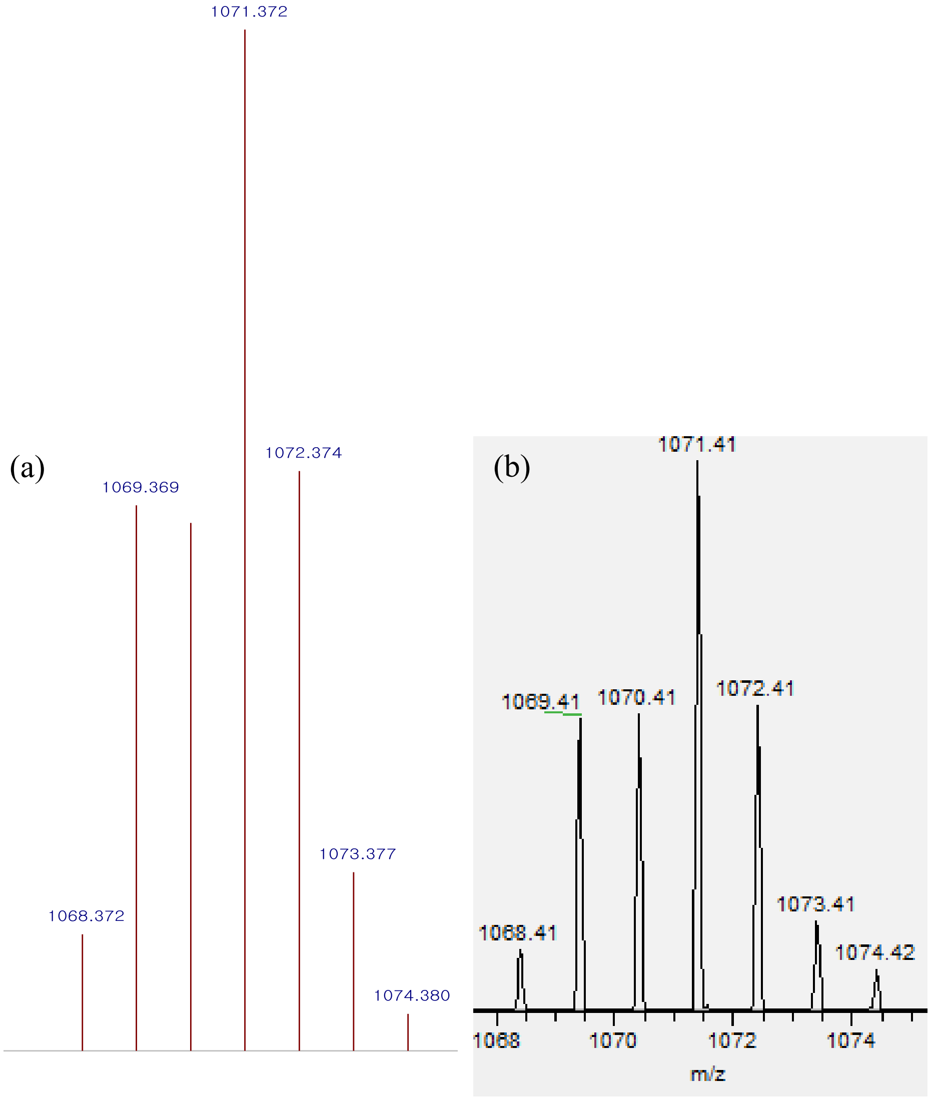

Fig. S32. Simulated (a) and experimental (b) isotropic distribution patterns of complex **8b**: the molecular ion peak ($[M - PF_6]^+$).

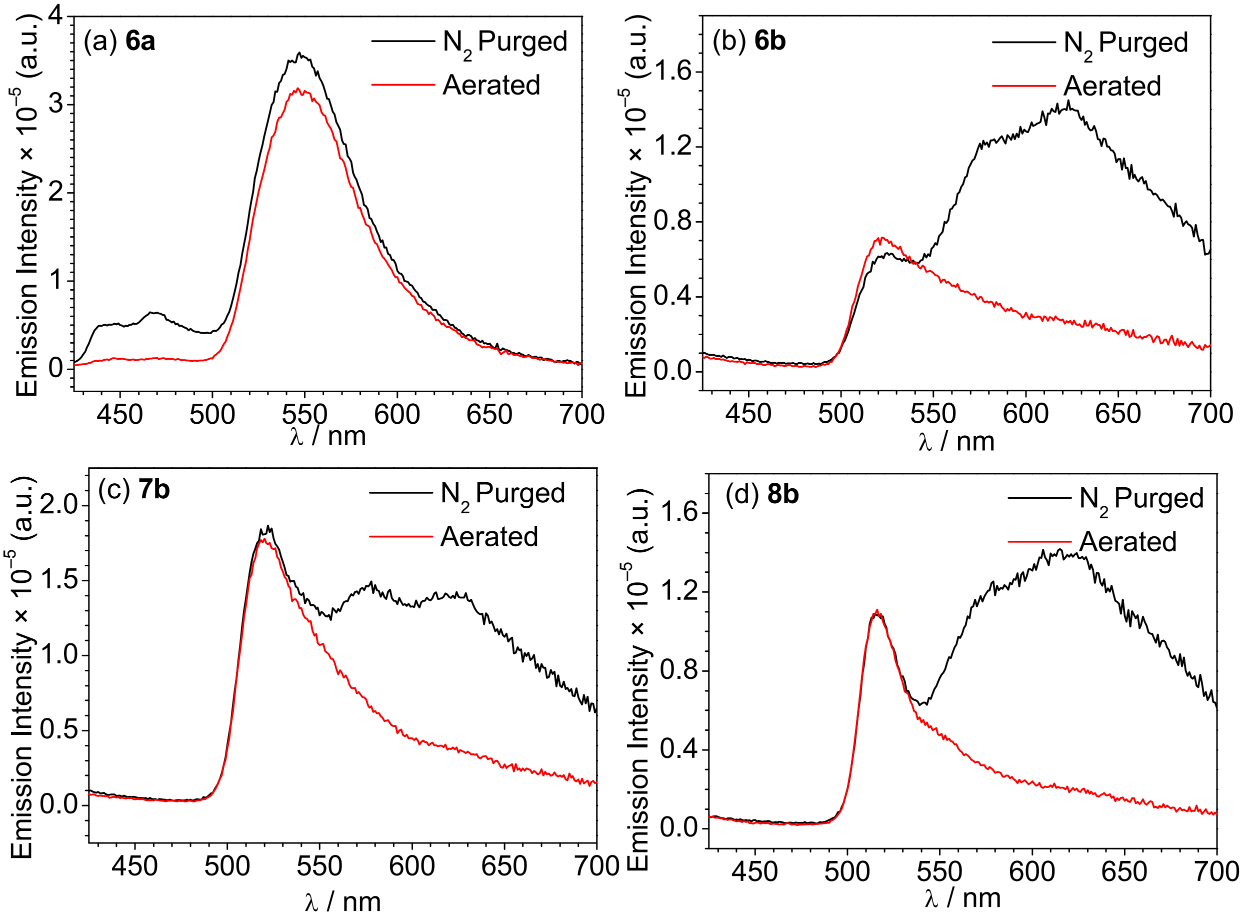

Fig. S33. Emission spectra of complexes **6a** (a), **6b** (b), **7b** (c), and **8b** (d), were measured at room temperature in CH_2Cl_2 under N_2-purged and aerated conditions (λ_{ex} = 310 nm).

References

1 J. Bartelmess, W. W. Weare, N. Latortue, C. Duong and D. S. Jones, *New J. Chem.*, 2013, **37**, 2663.

2 J. Bartelmess and W. W. Weare, *Dyes Pigments*, 2013, **97**, 1–8.

3 K. S. Choung and T. S. Teets, *ChemPhotoChem*, , DOI:10.1002/cptc.201800202.

4 H. Chong, E. Fron, Z. Liu, S. Boodts, J. Thomas, J. N. Harvey, J. Hofkens, W. Dehaen, M. Van der Auweraer and M. Smet, *Chem. - Eur. J.*, 2017, **23**, 4687–4699.

5 A. Maity, J. C. Kölsch, H. Na and T. S. Teets, *Dalton Trans.*, 2017, **46**, 11757–11767.

6 G. M. Sheldrick, *Acta Crystallogr. A*, 2008, **64**, 112–122.

7 A. L. Spek, *Acta Crystallogr. D Biol. Crystallogr.*, 2009, **65**, 148–155.
